# Supplementary material for: Molecular Evolutionary Dynamics of Energy Limited Microorganisms
Source: Mol Biol Evol. 2021 Jul 13;38(10):4532–45. doi: 10.1093/molbev/msab195 (PMC8476154; doi:10.1093/molbev/msab195)
Supplement: msab195_Supplementary_Data [file msab195_supplementary_data.zip › supplement_latex.zip!/supplement_latex/Phylo_Evol_Timeseries_supplement.pdf]

Supplementary Information: Molecular Evolutionary Dynamics of Energy-limited Microorganisms

William R. Shoemaker[1,2,*], Evgeniya Polezhaeva[1], Kenzie B. Givens[1, 3], and Jay T. Lennon[1,*]

[1]Department of Biology, Indiana University, Bloomington, IN, 47405, USA

[2]Current affiliation: Department of Ecology and Evolutionary Biology, University of California, Los Angeles, CA, 90095, USA

[3]Current affiliation: Luddy School of Informatics, Computing, and Engineering, Indiana University, Bloomington IN, 47408, USA

May 18, 2021

1 Variant calling

The first 20 bp of all reads were trimmed and all read pairs where at least one pair had a mean Phred quality less than 20 were removed `cutadept v1.9.1` [1]. Candidate variants were identified by modifying the codebase of a previously published approach under GPL v2 [2]. We provide a brief overview of the method used in Good et al. (2017) and all alterations made, though we direct

the reader to the original publication for an in-depth account of the pipeline. Using `breseq v0.32.0` [3] to align trimmed reads to the reference genome, we generated a list of candidate junctions for all samples. Candidate junctions were merged across temporal samples for each taxon using `gdtools`, which was then passed as an argument to a second round of `breseq` using `--user-evidence-gd`. Using `samtools mpileup` [4] and open access Python scripts from [2], we identified candidate SNVs and small indels from BAM files generated by the second `breseq` run. We defined trajectories of ordered pairs (A_{pmt}, D_{pmt}) for the alternative allele count and total depth of coverage for mutation m in sample t from population p. Alternative alleles for indels $<$ 100bp are merged as a single "compound" mutation trajectory. Indels $\geq$ 100bp are considered structural variants and are processed with candidate junctions identified by `breseq`.

Candidate junctions were processed using a custom Python script that merges similar candidate junctions from the same population into a single "compound" junction candidate. Each of these candidates was recorded as a single trajectory (A_{pmt}, D_{pmt}). We examined all candidate mutations with $A_{pmt} \geq 2$ in at least two samples and $D_{pmt} \geq 10$ in at least one samples, with an observed frequency $f_{pmt} \equiv A_{pmt}/D_{pmt} \geq 0.05$.

Statistical support of each candidate mutation was established using two summary statistics: 1) the autocorrelation of frequencies between timepoints (C^{*}) and 2) the derived allele sojourn weight (I). Both functions are described at length in Good et al. (2017). For our purposes here C^{*} can be described as a modified form of the autocorrelation function

$$C \equiv \sum_{t} \left(f_{t+1} - \overline{f}\right) \left(f_{t} - \overline{f}\right) \qquad (1)$$

where f_t is the frequency of the mutation at a given timepoint and $\overline{f}$ is the mean frequency over the entire timecourse. We use a modified form of this func-

tion that has the same purpose, but accounts for discreteness and uncertainty in f_t due to finite coverage.

The derived allele sojourn time is less intuitive. C^* treats positive and negative deviations from the mean the same way (i.e., symmetric). Real mutations can have low frequencies for extended periods of time. A large area under this allele frequency trajectory curve would suggest that this mutation is not an error. To capture this trend, the statistic in Good *et al.* (2017) examines runs of 2 or more timepoints where f_t is larger than a threshold frequency f^* for that entire run. The run with the largest value of

$$I = \sum_{t=t_1}^{t_2} = f_t - f^* \tag{2}$$

The value of f^* was chosen using the criteria in Good *et al.* to be as low as possible while allowing for error rates higher than $1/D_t$ (2017).

The two P-values were merged as a composite P-value using the following function:

$$T = \sum_k \theta\left(P^* - P_k\right) \log\left(\frac{1}{P_k}\right) \tag{3}$$

where $\theta(\cdot)$ is the Heaviside step function and $P^* = (0.05)^{1/2}$. Significance was assessed by calculating the null distribution of T for all mutations and nonsignificant mutations were removed from downstream analyses. We did not include the third statistic, the average frequency relaxation time, used in Good *et al.* due to fact that our experiment covers a relatively brief evolutionary timescale (3,000 generations vs. 60,000) (2017). We annotated all mutations as described in Good *et al.* (2017).

2 Mutation trajectory inference

We use the naive estimator $\hat{f}_{pmt} = A_{pmt}/D_{pmt}$ as our measure of mutation frequency. We examined mutation accumulation as

$$M(t) \equiv \sum_{m} \hat{f}_{pmt} \quad (4)$$

a measure that uses information from mutation frequencies in addition to the number of mutations in the population.

To infer whether a high frequency mutation is present in all individuals (i.e., "fixed"), we used the hidden Markov model introduced in Good *et al.* with $(A_{mt};D_{mt})$ as the observed sequence of emissions (2017). To briefly summarize this model, mutations start in an ancestral state **A** where $f_{mt} = 0$. At each timepoint mutations can transition to a polymorphic state **P** where the frequency remains as $0 < f_{mt} < 1$. From here the mutation can either transition to a fixed state **F** or go extinct **E**. **E** states are allowed to re-appear as **A**, though multiple mutations occurring at the same site is a rare event. The behavior of the HMM was fairly insensitive to the chosen initial transition probabilities, so we set the initial transition probabilities to those in Good *et al.* (2017).

3 Parallelism and divergence

We identified potential targets of selection by examining the distribution of nonsynonymous mutations across genes. The statistical framework of this approach was developed in Good *et al.* (Good et al., 2017). To briefly summarize, gene-level parallelism was assessed by calculating the *multiplicity* of each gene as

$$m_i = n_i \cdot \frac{\overline{L}}{L_i} \quad (5)$$

where n_i and L_i is the number of mutations observed and the length of the ith gene and $\overline{L}$ is the mean length of all genes. Under this definition, the null hypothesis is that all genes have the same multiplicity $\overline{m} = n_{tot}/N_{genes}$. Using the observed and expected values, we can quantify the net increase of the log-likelihood of the alternative hypothesis relative to the null

$$\Delta\ell = \sum_i n_i \log\left(\frac{m_i}{\overline{m}}\right) \tag{6}$$

where significance is assessed using permutation tests. Because this measure can be sensitive to n_{tot}, for comparisons across different strains and treatments we randomly sub-sampled mutations as a multinomial distribution, where the probability of sampling a mutation at gene i was given by $p_i = n_i/n_{tot}$. Multinomial sampling was performed 10,000 times with a sub-sampled n_{tot} set to 50.

To identify specific genes that are enriched for mutations, we calculated the P-value of each gene as

$$P_i = \sum_{n \geq n_i} \frac{\left(\frac{n_{tot} L_i}{\overline{L} N_{genes}}\right)^n}{n!} e^{-\frac{n_{tot} L_i}{\overline{L} N_{genes}}} \tag{7}$$

where FDR correction was performed by defining a critical P-value (P^*) based on the survival curve of a Poisson distribution, the null hypothesis (see Good et al., 2017 for additional details). We then defined the set of significant genes for each treatment-strain combination as:

$$I = \{i : P_i \leq P^*(\alpha)\} \tag{8}$$

for $\alpha = 0.05$.

References

[1] Marcel Martin. Cutadapt removes adapter sequences from high-throughput sequencing reads. *EMBnet.journal*, 17(1):10–12, May 2011.

[2] Benjamin H. Good, Michael J. McDonald, Jeffrey E. Barrick, Richard E. Lenski, and Michael M. Desai. The dynamics of molecular evolution over 60,000 generations. *Nature*, 551(7678):45–50, November 2017.

[3] Daniel E. Deatherage and Jeffrey E. Barrick. Identification of mutations in laboratory-evolved microbes from next-generation sequencing data using breseq. *Methods in Molecular Biology (Clifton, N.J.)*, 1151:165–188, 2014.

[4] Heng Li, Bob Handsaker, Alec Wysoker, Tim Fennell, Jue Ruan, Nils Homer, Gabor Marth, Goncalo Abecasis, Richard Durbin, and 1000 Genome Project Data Processing Subgroup. The Sequence Alignment/Map format and SAMtools. *Bioinformatics (Oxford, England)*, 25(16):2078–2079, August 2009.

PC	Transition	ρ^2	P
1	A : T $\rightarrow$ C : G	0.906	$< 10^{-4}$
	G : C $\rightarrow$ T : A	0.746	$< 10^{-4}$
	G : C $\rightarrow$ C : G	0.627	0.015
2	A : T $\rightarrow$ G : C	0.758	0.002
	G : C $\rightarrow$ A : T	0.657	0.015

Table S1: Factor loadings that are significantly correlated with the first and second components of the PCA visualized in Fig. 2.

Genus	Treatment	Locus tag	n_{mut} fixed	n_{mut} $f_{max} \geq 0.8$	Annotation
Bacillus	1-day	B4U62_RS00170	1	1	23S ribosomal RNA
Bacillus	1-day	B4U62_RS00390	1	1	stage II sporulation protein E
Bacillus	1-day	B4U62_RS00585	1	1	DNA integrity scanning protein DisA
Bacillus	1-day	B4U62_RS00685	1	1	DNA-directed RNA polymerase subunit beta
Bacillus	1-day	B4U62_RS01310	1	3	APC family permease
Bacillus	1-day	B4U62_RS01735	1	3	DUF475 domain-containing protein
Bacillus	1-day	B4U62_RS01940	1	1	Assimilatory nitrate reductase catalytic subunit
Bacillus	1-day	B4U62_RS01950	1	1	NarK/NasA family nitrate transporter
Bacillus	1-day	B4U62_RS02045	1	1	Surfactin biosynthesis thioesterase SrfAD
Bacillus	1-day	B4U62_RS02495	2	2	Divalent metal cation transporter
Bacillus	1-day	B4U62_RS02855	1	1	Endopeptidase
Bacillus	1-day	B4U62_RS03535	1	1	Tetratricopeptide repeat protein
Bacillus	1-day	B4U62_RS03840	1	1	Diacylglycerol kinase
Bacillus	1-day	B4U62_RS04785	1	1	Glycosyltransferase
Bacillus	1-day	B4U62_RS04835	1	1	Aromatic acid exporter family protein
Bacillus	1-day	B4U62_RS04860	1	1	Hypothetical protein
Bacillus	1-day	B4U62_RS04890	1	1	tRNA-Glu
Bacillus	1-day	B4U62_RS05145	1	1	Cold-shock protein
Bacillus	1-day	B4U62_RS05175	1	2	YhcN/YlaJ family sporulation lipoprotein
Bacillus	1-day	B4U62_RS05315	1	1	Citrate synthase
Bacillus	1-day	B4U62_RS05375	1	1	MerR family transcriptional regulator
Bacillus	1-day	B4U62_RS05430	2	2	Na+/H+ antiporter NhaC
Bacillus	1-day	B4U62_RS05510	1	1	Cation:proton antiporter
Bacillus	1-day	B4U62_RS05555	1	1	3'-5' exoribonuclease YhaM
Bacillus	1-day	B4U62_RS06285	1	1	Beta-ketoacyl synthase II
Bacillus	1-day	B4U62_RS06330	2	3	Peptide ABC transporter protein
Bacillus	1-day	B4U62_RS06345	1	1	ABC transporter ATP-binding protein
Bacillus	1-day	B4U62_RS06650	1	1	BglG family transcription antiterminator

Bacillus	1-day	B4U62_RS06735	1	1	DUF4309 domain-containing protein
Bacillus	1-day	B4U62_RS06775	1	1	Hypothetical protein
Bacillus	1-day	B4U62_RS07360	1	1	MerR family transcriptional regulator
Bacillus	1-day	B4U62_RS07420	1	1	DedA family protein
Bacillus	1-day	B4U62_RS07740	1	1	Chemotaxis protein CheV
Bacillus	1-day	B4U62_RS07750	1	2	MFS transporter
Bacillus	1-day	B4U62_RS08325	1	1	16S rRNA methyltransferase
Bacillus	1-day	B4U62_RS08410	1	1	Sigma-E processing peptidase SpoIIGAv
Bacillus	1-day	B4U62_RS08450	1	1	Cell division protein SepF
Bacillus	1-day	B4U62_RS08580	1	1	Calcium-translocating P-type ATPase
Bacillus	1-day	B4U62_RS08605	1	1	Bifunctional ligase CoaBC
Bacillus	1-day	B4U62_RS08680	1	1	L-serine ammonia-lyase subunit beta
Bacillus	1-day	B4U62_RS08785	1	1	Ribonuclease HII
Bacillus	1-day	B4U62_RS08870	1	1	Flagellar assembly protein FliH
Bacillus	1-day	B4U62_RS08950	1	1	Flagellar biosynthesis protein FlhB
Bacillus	1-day	B4U62_RS08955	1	1	Flagellar biosynthesis protein FlhA
Bacillus	1-day	B4U62_RS09075	1	1	Translation initiation factor IF-2
Bacillus	1-day	B4U62_RS09090	1	1	tRNA pseudouridine(55) synthase TruB
Bacillus	1-day	B4U62_RS09270	1	1	DNA mismatch repair protein MutS
Bacillus	1-day	B4U62_RS09345	1	1	Amino acid adenylation domain-containing protein
Bacillus	1-day	B4U62_RS09350	1	1	SDR family NAD(P)-dependent oxidoreductase
Bacillus	1-day	B4U62_RS09485	1	1	Transcriptional repressor GlnR
Bacillus	1-day	B4U62_RS09645	1	1	SRPBCC domain-containing protein
Bacillus	1-day	B4U62_RS09965	1	1	Amino acid adenylation domain-containing protein
Bacillus	1-day	B4U62_RS10230	1	1	DUF3221 domain-containing protein
Bacillus	1-day	B4U62_RS10440	1	1	GNAT family N-acetyltransferase
Bacillus	1-day	B4U62_RS11410	1	1	Hypothetical protein
Bacillus	1-day	B4U62_RS11595	1	1	Hypothetical protein
Bacillus	1-day	B4U62_RS12015	1	1	2-dehydro-3-deoxygluconokinase
Bacillus	1-day	B4U62_RS12140	1	1	Endonuclease III

Bacillus	1-day	B4U62_RS12150	1	1	Asparagine–tRNA ligase
Bacillus	1-day	B4U62_RS12315	1	1	Chorismate mutase
Bacillus	1-day	B4U62_RS12420	1	2	30S ribosomal protein S1
Bacillus	1-day	B4U62_RS12755	1	1	Transcriptional repressor
Bacillus	1-day	B4U62_RS12770	1	1	NAD-dependent malic enzyme
Bacillus	1-day	B4U62_RS13010	1	1	Multidrug efflux MFS transporter Bmr
Bacillus	1-day	B4U62_RS13070	1	1	Citrate synthase
Bacillus	1-day	B4U62_RS13110	3	3	Sporulation transcription factor Spo0A
Bacillus	1-day	B4U62_RS13470	1	1	Hypothetical protein
Bacillus	1-day	B4U62_RS13740	1	1	50S ribosomal protein L11 methyltransferase
Bacillus	1-day	B4U62_RS14145	1	1	Hypothetical protein
Bacillus	1-day	B4U62_RS14390	4	4	Cation transporter
Bacillus	1-day	B4U62_RS14670	1	2	TetR/AcrR family transcriptional regulator
Bacillus	1-day	B4U62_RS14745	1	1	Transcription elongation factor GreA
Bacillus	1-day	B4U62_RS14830	1	1	Hypothetical protein
Bacillus	1-day	B4U62_RS14905	2	2	Adenine phosphoribosyltransferase
Bacillus	1-day	B4U62_RS15030	1	1	Quinolinate synthase NadA
Bacillus	1-day	B4U62_RS15150	1	1	Valine–tRNA ligase
Bacillus	1-day	B4U62_RS15605	1	1	Putative sporulation protein YtxC
Bacillus	1-day	B4U62_RS15715	1	1	Pyruvate kinase
Bacillus	1-day	B4U62_RS15720	1	1	ATP-dependent 6-phosphofructokinase
Bacillus	1-day	B4U62_RS15770	2	2	CBS domain-containing protein
Bacillus	1-day	B4U62_RS15990	1	1	Acetate–CoA ligase
Bacillus	1-day	B4U62_RS16050	1	1	DNA translocase SftA
Bacillus	1-day	B4U62_RS16120	1	1	Hypothetical protein
Bacillus	1-day	B4U62_RS16215	1	1	Extracellular solute-binding protein
Bacillus	1-day	B4U62_RS16880	1	1	Bifunctional aldolase/short-chain dehydrogenase
Bacillus	1-day	B4U62_RS16995	1	1	Lrp/AsnC family transcriptional regulator
Bacillus	1-day	B4U62_RS17250	1	1	PucR family transcriptional regulator
Bacillus	1-day	B4U62_RS17290	1	1	2,3-dihydro-2,3-dihydroxybenzoate dehydrogenase

Bacillus	1-day	B4U62_RS17510	1	1	Purine permease
Bacillus	1-day	B4U62_RS17950	1	1	YvrJ family protein
Bacillus	1-day	B4U62_RS18090	1	1	Cadmium-translocating P-type ATPase
Bacillus	1-day	B4U62_RS18215	1	1	ATP-binding cassette domain-containing protein
Bacillus	1-day	B4U62_RS18230	1	1	Sporulation-delaying protein SdpB
Bacillus	1-day	B4U62_RS18305	1	2	2,3-bisphosphoglycerate phosphoglycerate mutase
Bacillus	1-day	B4U62_RS18430	1	1	Sugar ABC transporter permease
Bacillus	1-day	B4U62_RS18445	1	1	FadR family transcriptional regulator
Bacillus	1-day	B4U62_RS18495	2	2	Pyruvyl transferase
Bacillus	1-day	B4U62_RS18630	1	1	ATP-dependent Clp protease proteolytic subunit
Bacillus	1-day	B4U62_RS18815	1	1	Imidazoleglycerol-phosphate dehydratase HisB
Bacillus	1-day	B4U62_RS18945	1	1	Excinuclease ABC subunit UvrA
Bacillus	1-day	B4U62_RS18975	1	1	Serine protease
Bacillus	1-day	B4U62_RS19580	1	1	Hypothetical protein
Bacillus	1-day	B4U62_RS19840	1	1	F0F1 ATP synthase subunit beta
Bacillus	1-day	B4U62_RS20050	2	3	ABC transporter ATP-binding protein
Bacillus	1-day	B4U62_RS20225	1	1	MarR family transcriptional regulator
Bacillus	1-day	B4U62_RS20360	1	1	dTDP-4-dehydrorhamnose reductase
Bacillus	1-day	B4U62_RS20790	1	1	MFS transporter
Bacillus	1-day	B4U62_RS20905	1	1	YbhB/YbcL family Raf kinase inhibitor-like protein
Bacillus	1-day	B4U62_RS20970	1	1	GntP family permease
Bacillus	1-day	B4U62_RS21155	1	1	Histidine ammonia-lyase
Bacillus	1-day	B4U62_RS21195	1	1	Sugar-binding transcriptional regulator
Bacillus	1-day	B4U62_RS21335	1	1	MFS transporter
Bacillus	1-day	B4U62_RS21795	1	1	DUF2232 domain-containing protein
Bacillus	1-day	B4U62_RS22065	1	1	16S rRNA methyltransferase RsmG
Bacillus	1-day	B4U62_RS22410	1	1	Replicative DNA helicase
Bacillus	10-day	B4U62_RS06875	1	1	ATP-dependent metallopeptidase FtsH/Yme1/Tma
Bacillus	10-day	B4U62_RS09275	1	2	DNA mismatch repair endonuclease MutL
Bacillus	10-day	B4U62_RS12480	1	1	LysM peptidoglycan-binding protein

Bacillus	10-day	B4U62_RS13565	1	2	YitT family protein
Bacillus	10-day	B4U62_RS14390	1	2	Cation transporter
Bacillus	10-day	B4U62_RS15625	1	1	Transcriptional regulator NrdR
Bacillus	100-day	B4U62_RS08860	1	1	Flagellar basal body M-ring protein FliF
Deinococcus	1-day	DR_0403	1	2	Inosine-uridine preferring nucleoside hydrolase
Deinococcus	1-day	DR_0544	1	1	Hypothetical protein
Deinococcus	1-day	DR_0958	1	2	Peptide ABC transporter permease
Deinococcus	1-day	DR_0986	1	1	Extracellular solute-binding protein
Deinococcus	1-day	DR_1670	1	2	Cysteinyl-tRNA synthetase
Deinococcus	1-day	DR_2024	1	1	Hypothetical protein
Deinococcus	1-day	DR_2033	1	3	Glutamine synthase
Deinococcus	1-day	DR_2168	1	2	16S rRNA m5C967 methyltransferase
Deinococcus	1-day	DR_2286	1	1	Hypothetical protein
Deinococcus	1-day	DR_A0283	1	2	Serine protease
Deinococcus	10-day	DR_0008	1	1	Hypothetical protein
Deinococcus	10-day	DR_0167	1	1	Hypothetical protein
Deinococcus	10-day	DR_0198	1	1	Recombination protein RecR
Deinococcus	10-day	DR_0349	1	1	ATP-dependent protease LA
Deinococcus	10-day	DR_0613	1	1	Hypothetical protein
Deinococcus	10-day	DR_0698	1	1	v-type ATP synthase subunit C
Deinococcus	10-day	DR_0877	1	1	Hypothetical protein
Deinococcus	10-day	DR_0878	4	4	Adenine phosphoribosyltransferase
Deinococcus	10-day	DR_0879	2	2	Hypothetical protein
Deinococcus	10-day	DR_0907	1	1	CSD family cold shock protein
Deinococcus	10-day	DR_0912	3	3	DNA-directed RNA polymerase subunit beta
Deinococcus	10-day	DR_0932	1	1	Polyprenyl synthase
Deinococcus	10-day	DR_1089	1	1	Recombination protein F
Deinococcus	10-day	DR_1229	1	1	Hypothetical protein
Deinococcus	10-day	DR_1237	1	1	Hypothetical protein
Deinococcus	10-day	DR_1352	1	1	Dihydrolipoamide acetyltransferase-like protein

Deinococcus	10-day	DR_1501	1	1	NADH dehydrogenase I subunit E
Deinococcus	10-day	DR_1514	1	1	2-phosphoglycerate kinase
Deinococcus	10-day	DR_1567	1	1	Peptide ABC transporter ATP-binding protein
Deinococcus	10-day	DR_1568	1	1	Peptide ABC transporter ATP-binding protein
Deinococcus	10-day	DR_1597	1	1	Glucose 6-phosphate dehydrogenase assembly protein Op
Deinococcus	10-day	DR_1705	1	1	Hydrolase family protein
Deinococcus	10-day	DR_1707	1	1	DNA-directed DNA polymerase
Deinococcus	10-day	DR_1976	1	1	DNA mismatch repair protein MutS
Deinococcus	10-day	DR_2059	1	1	Glycyl-tRNA synthetase
Deinococcus	10-day	DR_2134	1	1	ABC transporter ATP-binding protein
Deinococcus	10-day	DR_2224	1	1	Tellurium resistance protein TerZ
Deinococcus	10-day	DR_2234	1	1	Hypothetical protein
Deinococcus	10-day	DR_2248	1	1	Dipeptidyl peptidase IV-like protein
Deinococcus	10-day	DR_2370	1	1	Dihydrolipoamide dehydrogenase E3 component
Deinococcus	10-day	DR_2583	1	1	Malate dehydrogenase
Deinococcus	10-day	DR_B0051	2	2	Hypothetical protein
Deinococcus	10-day	DR_B0052	1	1	Hypothetical protein
Deinococcus	10-day	DR_C0007	1	1	Hypothetical protein
Deinococcus	10-day	DR_C0008	1	1	Hypothetical protein
Deinococcus	10-day	DR_r09	1	1	23S ribosomal RNA
Deinococcus	100-day	DR_1301	1	1	Hypothetical protein
Deinococcus	100-day	DR_A0073	1	1	Cation-transporting P-type ATPase
Caulobacter	1-day	CCNA_00028	4	6	TonB-dependent receptor
Caulobacter	1-day	CCNA_00099	1	1	FtsZ-binding protein FzlC
Caulobacter	1-day	CCNA_01011	1	1	Imidazolonepropionase
Caulobacter	1-day	CCNA_01393	1	2	Soluble lytic murein transglycosylase
Caulobacter	1-day	CCNA_01556	1	1	LacI-family transcriptional regulator
Caulobacter	1-day	CCNA_01816	1	1	Nitrogen regulation protein ntrY
Caulobacter	1-day	CCNA_03177	1	1	Methylmalonyl-CoA mutase MeaA-like protein
Caulobacter	1-day	CCNA_03879	1	1	Uroporphyrinogen decarboxylase

Caulobacter	10-day	CCNA_00083	1	1	Phosphoglucomutase/phosphomannomutase
Caulobacter	10-day	CCNA_00313	1	1	Peptidase, M14 family
Caulobacter	10-day	CCNA_00798	1	1	ABC transporter, ATP-binding protein cydC
Caulobacter	10-day	CCNA_00851	1	2	Periplasmic multidrug efflux lipoprotein precursor
Caulobacter	10-day	CCNA_01395	1	1	Flavoprotein-ubiquinone oxidoreductase FzeA
Caulobacter	10-day	CCNA_02039	2	2	ATP-binding subunit ClpX
Caulobacter	10-day	CCNA_02157	1	1	HipB-family transcriptional regulator
Caulobacter	10-day	CCNA_03878	1	5	Ferrochelatase
Caulobacter	100-day	CCNA_02867	1	1	Phage tail length tape measure-related protein
Caulobacter	100-day	CCNA_03123	2	5	ArsR-family transcriptional regulator
Caulobacter	100-day	CCNA_03125	2	3	Melibiose carrier protein
Caulobacter	100-day	CCNA_03243	1	1	NADP+-dependent dehydrogenase
Janthinobacterium	100-day	FFI39_RS05855	1	1	Cell division protein ZapD
Janthinobacterium	100-day	FFI39_RS10710	1	1	Flagellar transcriptional regulator FlhC
Janthinobacterium	100-day	FFI39_RS13510	1	1	Efflux RND transporter
Janthinobacterium	100-day	FFI39_RS18515	1	3	Response regulator transcription factor
Pedobacter	1-day	FFJ24_RS00155	1	1	SDR family NAD(P)-dependent oxidoreductase
Pedobacter	1-day	FFJ24_RS00415	1	1	Hypothetical protein
Pedobacter	1-day	FFJ24_RS00495	2	2	TonB-dependent siderophore receptor
Pedobacter	1-day	FFJ24_RS00550	1	1	LptF/LptG family permease
Pedobacter	1-day	FFJ24_RS00930	1	1	Family 43 glycosylhydrolase
Pedobacter	1-day	FFJ24_RS00995	1	1	Endo-1,4-beta-xylanase
Pedobacter	1-day	FFJ24_RS01675	1	1	Glycoside hydrolase
Pedobacter	1-day	FFJ24_RS02155	1	1	GntR family transcriptional regulator
Pedobacter	1-day	FFJ24_RS02245	1	1	DNA repair protein RadC
Pedobacter	1-day	FFJ24_RS02305	1	1	Hypothetical protein
Pedobacter	1-day	FFJ24_RS02465	1	1	DNA-directed RNA polymerase subunit beta
Pedobacter	1-day	FFJ24_RS02835	1	1	DUF695 domain-containing protein
Pedobacter	1-day	FFJ24_RS03050	1	1	Hypothetical protein
Pedobacter	1-day	FFJ24_RS03145	1	1	Imidazole glycerol phosphate synthase subunit HisF

Pedobacter	1-day	FFJ24_RS03420	1	1	SusC/RagA family TonB-linked outer membrane protein
Pedobacter	1-day	FFJ24_RS03975	1	1	Aspartate aminotransferase family protein
Pedobacter	1-day	FFJ24_RS04035	1	1	Glycosyltransferase
Pedobacter	1-day	FFJ24_RS04060	1	1	Response regulator
Pedobacter	1-day	FFJ24_RS04100	1	1	FtsX-like permease family protein
Pedobacter	1-day	FFJ24_RS04310	1	1	Glycosyltransferase family 9 protein
Pedobacter	1-day	FFJ24_RS05090	1	1	Hypothetical protein
Pedobacter	1-day	FFJ24_RS05445	1	1	Ferritin
Pedobacter	1-day	FFJ24_RS05455	3	4	Hypothetical protein
Pedobacter	1-day	FFJ24_RS05480	1	1	Hypothetical protein
Pedobacter	1-day	FFJ24_RS05605	1	1	Hypothetical protein
Pedobacter	1-day	FFJ24_RS06370	1	1	Metallophosphoesterase
Pedobacter	1-day	FFJ24_RS06605	1	1	Glycoside hydrolase family 92 protein
Pedobacter	1-day	FFJ24_RS07700	1	1	Bcr/CflA family efflux MFS transporter
Pedobacter	1-day	FFJ24_RS07770	1	1	Hydrolase
Pedobacter	1-day	FFJ24_RS08480	1	1	Hypothetical protein
Pedobacter	1-day	FFJ24_RS08640	1	1	Glutamate synthase large subunit
Pedobacter	1-day	FFJ24_RS08735	1	1	Substrate-binding domain-containing protein
Pedobacter	1-day	FFJ24_RS08820	1	1	Replicative DNA helicase
Pedobacter	1-day	FFJ24_RS08870	1	1	S8 family serine peptidase
Pedobacter	1-day	FFJ24_RS08915	1	2	Glucose-6-phosphate dehydrogenase
Pedobacter	1-day	FFJ24_RS09040	3	3	TonB-dependent receptor
Pedobacter	1-day	FFJ24_RS09195	1	1	DUF4959 domain-containing protein
Pedobacter	1-day	FFJ24_RS09365	1	1	Phosphate acetyltransferase
Pedobacter	1-day	FFJ24_RS09730	1	1	Glycosyltransferase
Pedobacter	1-day	FFJ24_RS10145	1	1	Hypothetical protein
Pedobacter	1-day	FFJ24_RS11945	1	1	Efflux RND transporter permease subunit
Pedobacter	1-day	FFJ24_RS12005	1	1	Hypothetical protein
Pedobacter	1-day	FFJ24_RS12035	1	1	Hypothetical protein
Pedobacter	1-day	FFJ24_RS12235	2	2	DNA polymerase IV

Pedobacter	1-day	FFJ24_RS12250	1	1	Crp/Fnr family transcriptional regulator
Pedobacter	1-day	FFJ24_RS12770	1	1	SusC/RagA family TonB-linked protein
Pedobacter	1-day	FFJ24_RS12900	1	1	Anthranilate phosphoribosyltransferase
Pedobacter	1-day	FFJ24_RS14800	1	1	Tetratricopeptide repeat protein
Pedobacter	1-day	FFJ24_RS14945	1	1	Biopolymer transporter ExbD
Pedobacter	1-day	FFJ24_RS15010	1	1	DUF808 family protein
Pedobacter	1-day	FFJ24_RS15120	1	1	T9SS type B sorting protein
Pedobacter	1-day	FFJ24_RS15465	1	1	Alpha/beta fold hydrolase
Pedobacter	1-day	FFJ24_RS15515	1	1	Amino acid permease
Pedobacter	1-day	FFJ24_RS15765	1	1	TonB-dependent receptor
Pedobacter	1-day	FFJ24_RS16325	1	1	Response regulator
Pedobacter	1-day	FFJ24_RS16620	1	1	Hypothetical protein
Pedobacter	1-day	FFJ24_RS17420	1	1	DNA-directed RNA polymerase subunit alpha
Pedobacter	1-day	FFJ24_RS17480	1	1	50S ribosomal protein L6
Pedobacter	1-day	FFJ24_RS17705	1	1	Sensor histidine kinase
Pedobacter	1-day	FFJ24_RS18050	1	1	TraR/DksA family transcriptional regulator
Pedobacter	1-day	FFJ24_RS18195	1	1	N-acetylglucosamine kinase
Pedobacter	1-day	FFJ24_RS18645	1	1	Cupin-like domain-containing protein
Pedobacter	1-day	FFJ24_RS18690	1	1	Metallophosphoesterase
Pedobacter	1-day	FFJ24_RS19145	1	1	Type IX secretion system PorV
Pedobacter	1-day	FFJ24_RS19365	1	1	UMP kinase
Pedobacter	1-day	FFJ24_RS19400	1	1	Outer membrane beta-barrel protein
Pedobacter	1-day	FFJ24_RS19550	1	1	23S rRNA pseudouridine(2604) synthase RluF
Pedobacter	1-day	FFJ24_RS19635	1	1	PAS domain S-box protein
Pedobacter	1-day	FFJ24_RS19660	2	2	Undecaprenyl-phosphate glucose phosphotransferase
Pedobacter	1-day	FFJ24_RS20015	1	1	DUF3109 family protein
Pedobacter	1-day	FFJ24_RS20170	1	1	Oligosaccharide flippase family protein
Pedobacter	1-day	FFJ24_RS20285	1	1	Hypothetical protein
Pedobacter	1-day	FFJ24_RS20380	1	1	Superoxide dismutase
Pedobacter	1-day	FFJ24_RS21080	1	1	SusC/RagA family TonB-linked protein

Pedobacter	1-day	FFJ24_RS21100	1	1	SusD/RagB family nutrient-binding lipoprotein
Pedobacter	1-day	FFJ24_RS21105	3	3	SusC/RagA family TonB-linked protein
Pedobacter	1-day	FFJ24_RS21215	1	1	Hypothetical protein
Pedobacter	1-day	FFJ24_RS21495	1	1	AAA family ATPase
Pedobacter	1-day	FFJ24_RS22585	1	1	GHKL domain-containing protein
Pedobacter	1-day	FFJ24_RS22905	1	1	Type VI secretion system protein Hcp
Pedobacter	1-day	FFJ24_RS22945	2	2	Histidine ammonia-lyase
Pedobacter	1-day	FFJ24_RS23285	1	1	HAD-IB family phosphatase
Pedobacter	1-day	FFJ24_RS23720	1	1	TonB family protein
Pedobacter	1-day	FFJ24_RS24340	1	1	Lauroyl acyltransferase
Pedobacter	1-day	FFJ24_RS24430	1	1	Fumarate hydratase
Pedobacter	1-day	FFJ24_RS25035	1	1	TonB-dependent receptor
Pedobacter	1-day	FFJ24_RS25180	1	1	Hypothetical protein
Pedobacter	1-day	FFJ24_RS25285	1	1	DUF2723 domain-containing protein
Pedobacter	10-day	FFJ24_RS02470	1	1	DNA-directed RNA polymerase subunit beta
Pedobacter	10-day	FFJ24_RS07475	1	1	PAS domain S-box protein
Pedobacter	10-day	FFJ24_RS12525	1	1	SusC/RagA family TonB-linked protein
Pedobacter	10-day	FFJ24_RS14495	1	1	RluA family pseudouridine synthase
Pedobacter	10-day	FFJ24_RS14735	1	1	RelA/SpoT family protein
Pedobacter	10-day	FFJ24_RS17085	1	1	Acetate–CoA ligase
Pedobacter	10-day	FFJ24_RS18905	1	1	WYL domain-containing protein
Pedobacter	10-day	FFJ24_RS25295	1	1	Ribose-phosphate pyrophosphokinase
Pseudomonas	1-day	FFI16_RS00020	1	1	Hypothetical protein
Pseudomonas	1-day	FFI16_RS00110	1	1	Penicillin-binding protein
Pseudomonas	1-day	FFI16_RS00275	1	1	Response regulator
Pseudomonas	1-day	FFI16_RS01100	1	1	Ketol-acid reductoisomerase
Pseudomonas	1-day	FFI16_RS01890	1	1	Protein translocase subunit SecD
Pseudomonas	1-day	FFI16_RS02020	1	1	2-isopropylmalate synthase
Pseudomonas	1-day	FFI16_RS03235	1	1	Methyltransferase domain-containing protein
Pseudomonas	1-day	FFI16_RS04725	1	1	2-methylaconitate cis-trans isomerase PrpF

Pseudomonas	1-day	FFI16_RS05035	1	1	Nitrate reduction transcription regulator Fnr
Pseudomonas	1-day	FFI16_RS05325	1	1	Mechanosensitive ion channel
Pseudomonas	1-day	FFI16_RS06520	1	1	RraA family protein
Pseudomonas	1-day	FFI16_RS07235	1	1	Ribokinase
Pseudomonas	1-day	FFI16_RS08175	1	1	Fimbria/pilus outer membrane usher protein
Pseudomonas	1-day	FFI16_RS08470	1	1	FtsX-like permease family protein
Pseudomonas	1-day	FFI16_RS09450	1	1	ABC transporter permease
Pseudomonas	1-day	FFI16_RS09640	1	2	Hemagglutinin N-terminal domain-containing protein
Pseudomonas	1-day	FFI16_RS10080	1	1	Nuclear transport factor 2 family protein
Pseudomonas	1-day	FFI16_RS11460	1	1	C-type cytochrome
Pseudomonas	1-day	FFI16_RS12840	1	1	Ribonucleotide-diphosphate reductase subunit beta
Pseudomonas	1-day	FFI16_RS13190	1	1	Hypothetical protein
Pseudomonas	1-day	FFI16_RS14060	1	1	Precorrin-4 C(11)-methyltransferase
Pseudomonas	1-day	FFI16_RS15085	1	1	Zinc-binding dehydrogenase
Pseudomonas	1-day	FFI16_RS15585	1	1	UvrY/SirA/GacA family transcription factor
Pseudomonas	1-day	FFI16_RS15740	1	1	PAS domain S-box protein
Pseudomonas	1-day	FFI16_RS16780	1	1	Dipeptidase
Pseudomonas	1-day	FFI16_RS17515	1	1	DEAD/DEAH box helicase
Pseudomonas	1-day	FFI16_RS18145	1	1	Phage tail protein
Pseudomonas	1-day	FFI16_RS19225	1	1	L-threonine dehydrogenase
Pseudomonas	1-day	FFI16_RS19430	1	1	4-carboxymuconolactone decarboxylase
Pseudomonas	1-day	FFI16_RS20030	1	1	Methyltransferase domain-containing protein
Pseudomonas	1-day	FFI16_RS22765	1	1	Ferrochelatase
Pseudomonas	1-day	FFI16_RS23125	1	1	Gluconate transporter
Pseudomonas	1-day	FFI16_RS23555	1	1	Methyl-accepting chemotaxis protein
Pseudomonas	1-day	FFI16_RS24090	1	2	DNA mismatch repair endonuclease MutL
Pseudomonas	1-day	FFI16_RS24375	2	2	Bifunctional adenylyltransferase
Pseudomonas	1-day	FFI16_RS26360	1	1	Catalase HPII
Pseudomonas	1-day	FFI16_RS27220	1	2	Phosphate signaling complex protein PhoU
Pseudomonas	1-day	FFI16_RS27470	1	2	tonB-system energizer ExbB

Pseudomonas	1-day	FFI16_RS27910	1	2	Exopolyphosphatase
Pseudomonas	1-day	FFI16_RS27965	1	1	Transcription termination factor Rho
Pseudomonas	1-day	FFI16_RS29190	1	1	3-keto-5-aminohexanoate cleavage protein
Pseudomonas	1-day	FFI16_RS29735	1	1	Autotransporter domain-containing protein
Pseudomonas	1-day	FFI16_RS29785	1	1	SDR family NAD(P)-dependent oxidoreductase
Pseudomonas	10-day	FFI16_RS27875	1	2	C-type cytochrome

Table S2: The number of fixed nonsynonymous mutations and number of nonsynonymous mutations that reached a frequency of at least 0.8 within each gene for all taxa and treatments. Only genes with at least one fixed mutation are included.

Genus	Treatment intersection	Size	P
Bacillus	$1 \cap 10$	34	$< 10^{-3}$
	$1 \cap 100$	37	$< 10^{-3}$
	$10 \cap 100$	42	$< 10^{-3}$
	$1 \cap 10 \cap 100$	31	$< 10^{-3}$
Caulobacter	$1 \cap 10$	134	$< 10^{-3}$
	$1 \cap 100$	145	$< 10^{-3}$
	$10 \cap 100$	173	$< 10^{-3}$
	$1 \cap 10 \cap 100$	112	$< 10^{-3}$
Deinococcus	$1 \cap 10$	62	$< 10^{-3}$
	$1 \cap 100$	63	$< 10^{-3}$
	$10 \cap 100$	61	$< 10^{-3}$
	$1 \cap 10 \cap 100$	47	$< 10^{-3}$
Pedobacter	$1 \cap 10$	26	$< 10^{-3}$
	$1 \cap 100$	18	$< 10^{-3}$
	$10 \cap 100$	21	$< 10^{-3}$
	$1 \cap 10 \cap 100$	16	$< 10^{-3}$
Janthinobacterium	$1 \cap 100$	72	$< 10^{-3}$
Pseudomonas	$1 \cap 10$	76	$< 10^{-3}$
	$1 \cap 100$	136	$< 10^{-3}$
	$10 \cap 100$	135	$< 10^{-3}$
	$1 \cap 10 \cap 100$	70	$< 10^{-3}$

Table S3: The number of genes that were enriched for a given treatment intersection. P-values correspond to tests of whether a given intersection size is greater than expected by chance.

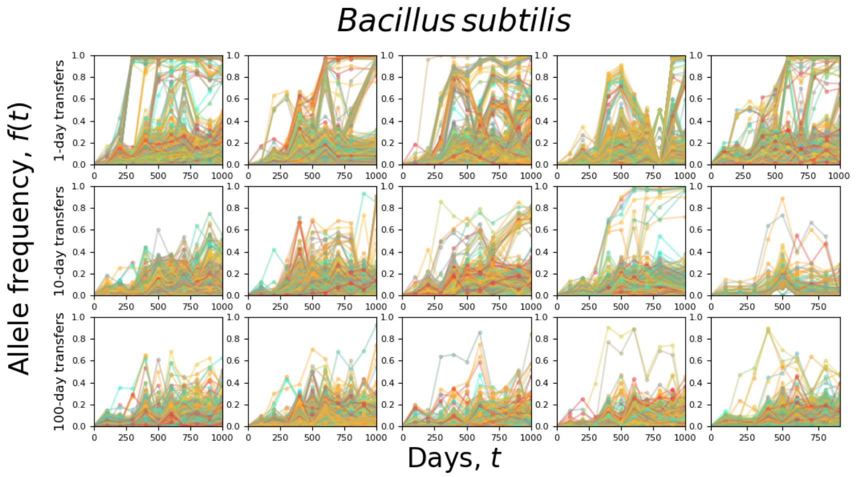

Figure S1: Allele frequency trajectories of all *Bacillus subtilis* replicate populations.

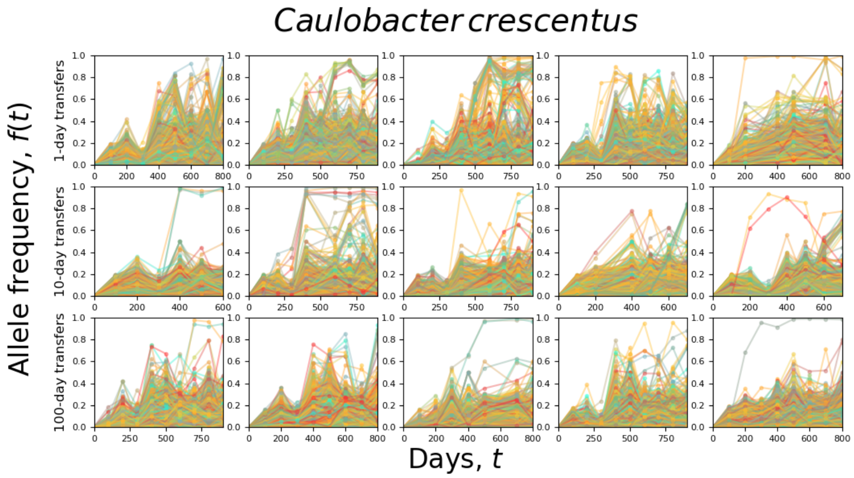

Figure S2: Allele frequency trajectories of all *Caulobacter crescentus* replicate populations.

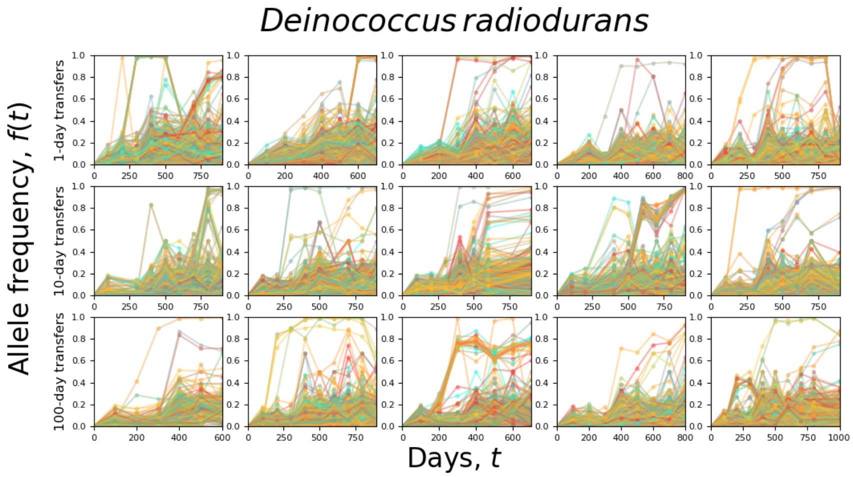

Figure S3: Allele frequency trajectories of all *Deinococcus radiodurans* replicate populations.

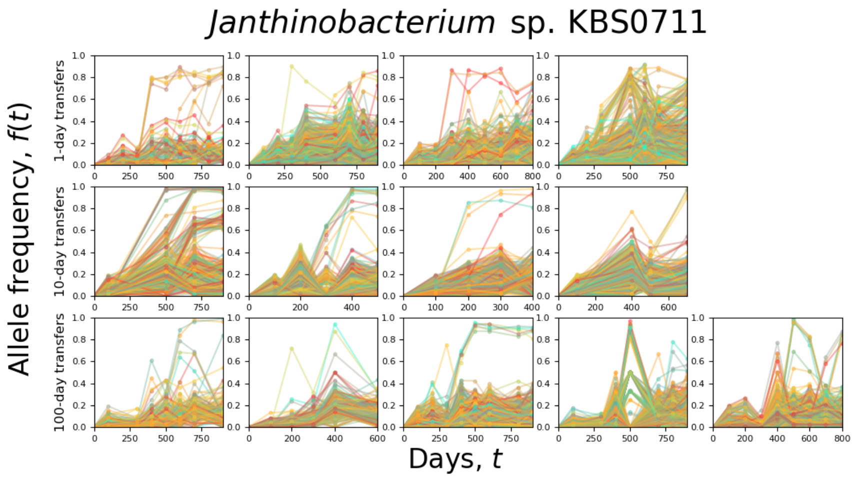

Figure S4: Allele frequency trajectories of all *Janthinobacterium* sp. KBS0711 replicate populations. Three 10-day replicate populations repeatedly went extinct over the course of the experiment, limiting the number of timepoints and preventing us from inferring mutation trajectories.

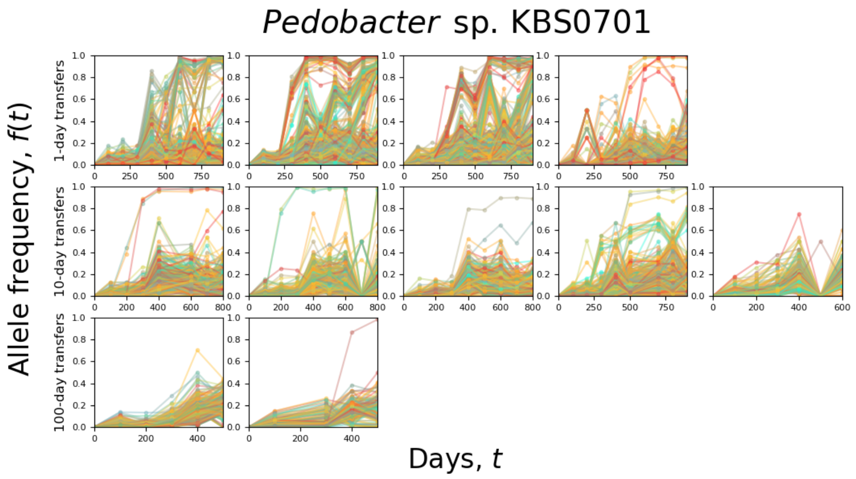

Figure S5: Allele frequency trajectories of all *Pedobacter* sp. KBS0701 replicate populations. Three 100-day replicate populations repeatedly went extinct over the course of the experiment, limiting the number of timepoints and preventing us from inferring mutation trajectories.

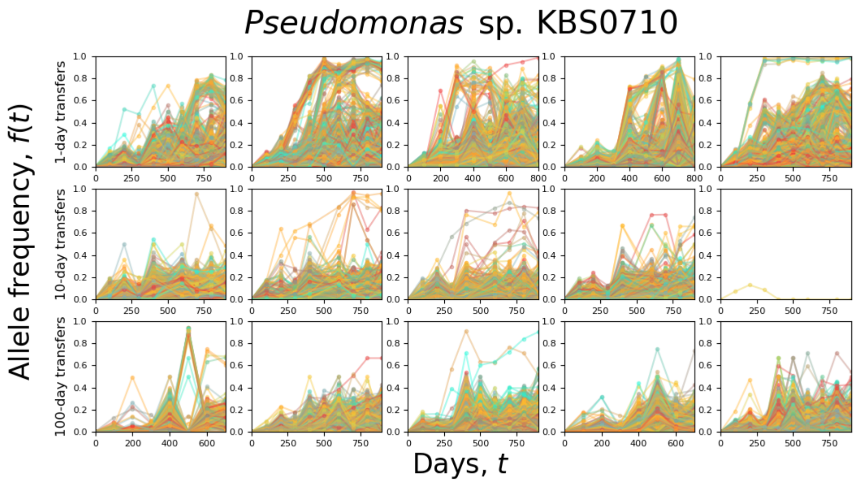

Figure S6: Allele frequency trajectories of all *Pseudomonas sp. KBS0710* replicate populations. Two 100-day replicate populations repeatedly went extinct over the course of the experiment, limiting the number of timepoints and preventing us from inferring mutation trajectories.

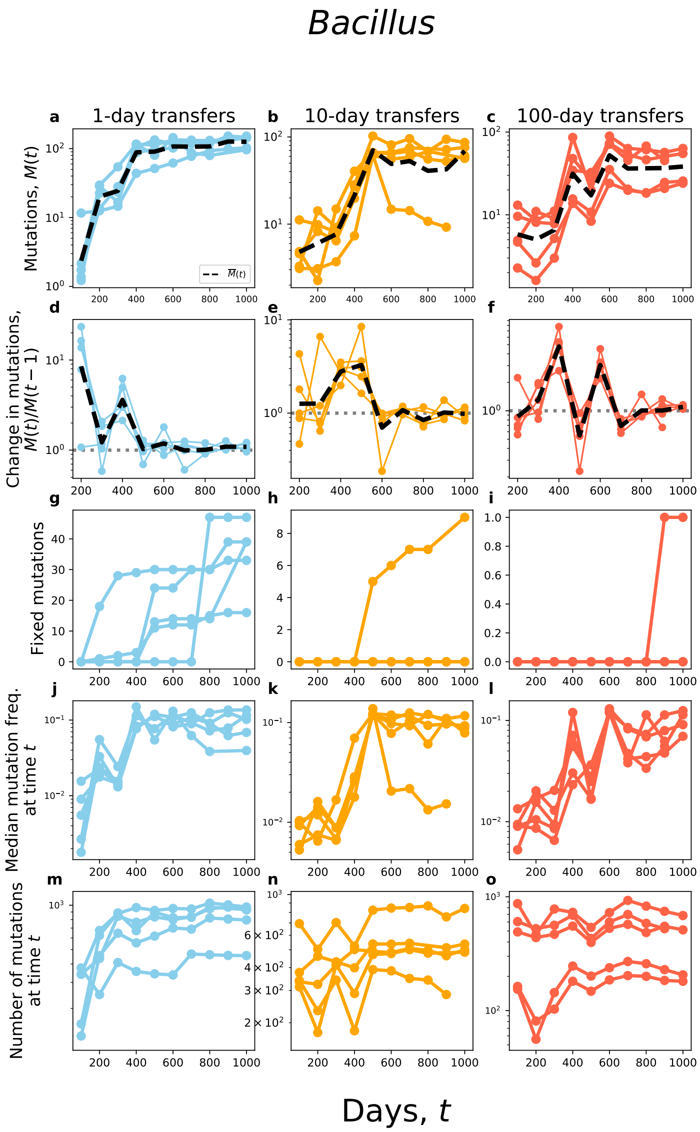

Figure S7: Molecular evolutionary dynamics of *Bacillus subtilis*. **a-c)** Cumulative mutation ($M(t)$) trajectories for both strains over time. The dashed black line is the mean. **d-f)** The change in $M(t)$ between timepoints across treatments. **g-i)** The cumulative number of fixed mutations over time for all populations within a given treatment. **j-l)** The median mutation frequency over time. **m-o)** The total number of polymorphic mutations segregating in the population over time.

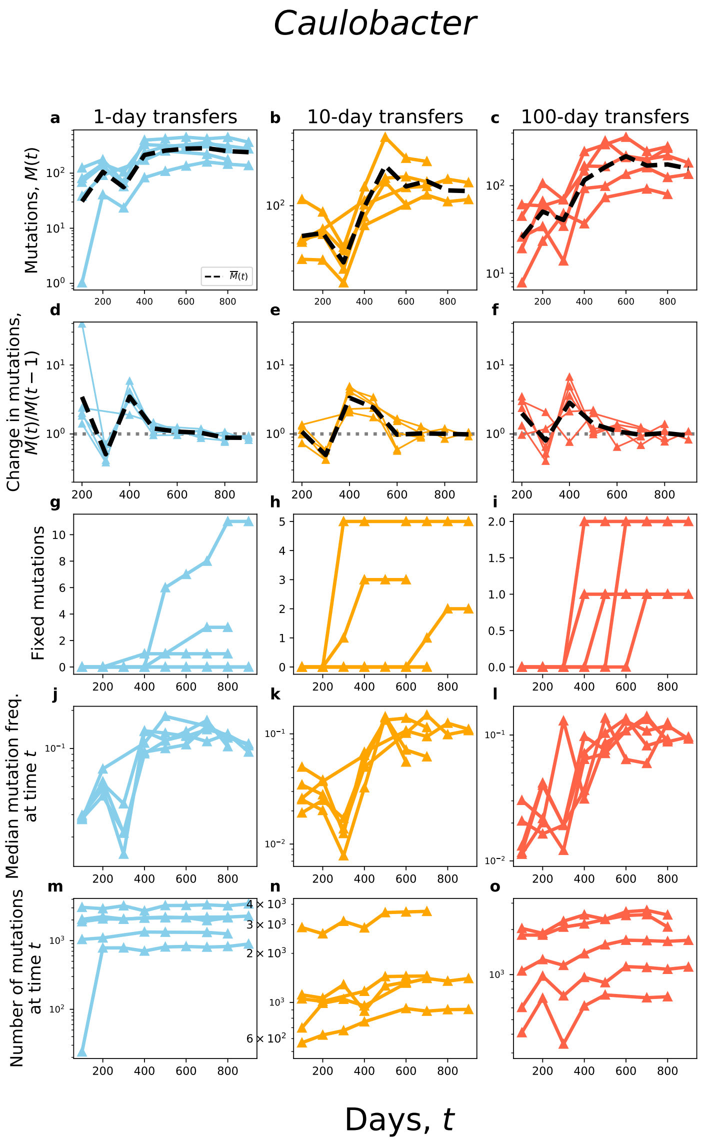

Figure S8: Molecular evolutionary dynamics of *Caulobacter crescentus*. **a-c)** Cumulative mutation ($M(t)$) trajectories for both strains over time. The dashed black line is the mean. **d-f)** The change in $M(t)$ between timepoints across treatments. **g-i)** The cumulative number of fixed mutations over time for all populations within a given treatment. **j-l)** The median mutation frequency over time. **m-o)** The total number of polymorphic mutations segregating in the population over time.

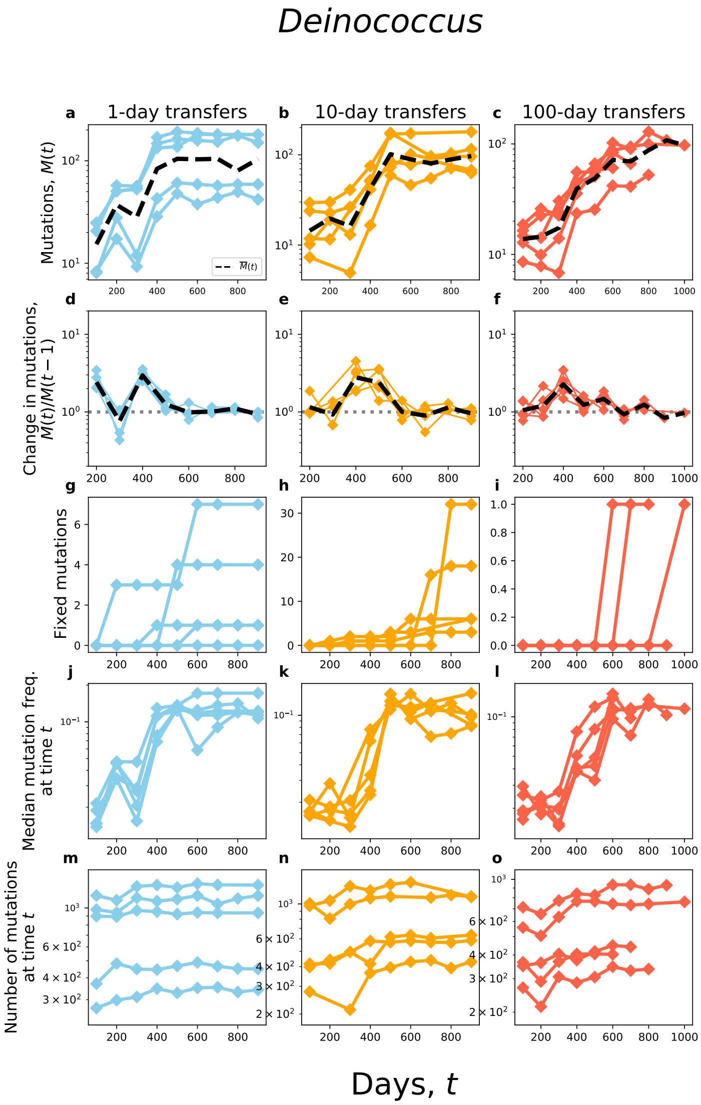

Figure S9: Molecular evolutionary dynamics of *Deinococcus radiodurans*. **a-c)** Cumulative mutation ($M(t)$) trajectories for both strains over time. The dashed black line is the mean. **d-f)** The change in $M(t)$ between timepoints across treatments. **g-i)** The cumulative number of fixed mutations over time for all populations within a given treatment. **j-l)** The median mutation frequency over time. **m-o)** The total number of polymorphic mutations segregating in the population over time.

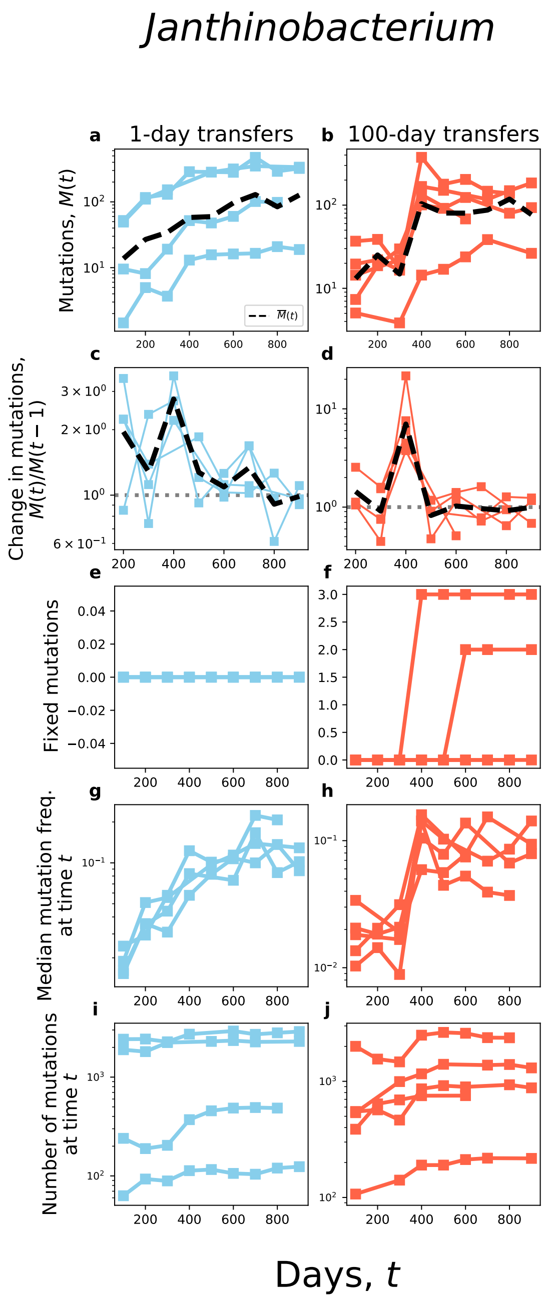

Figure S10: Molecular evolutionary dynamics of *Janthinobacterium* sp. KBS0711. **a-c)** Cumulative mutation ($M(t)$) trajectories for both strains over time. The dashed black line is the mean. **d-f)** The change in $M(t)$ between timepoints across treatments. **g-i)** The cumulative number of fixed mutations over time for all populations within a given treatment. **j-l)** The median mutation frequency over time. **m-o)** The total number of polymorphic mutations segregating in the population over time.

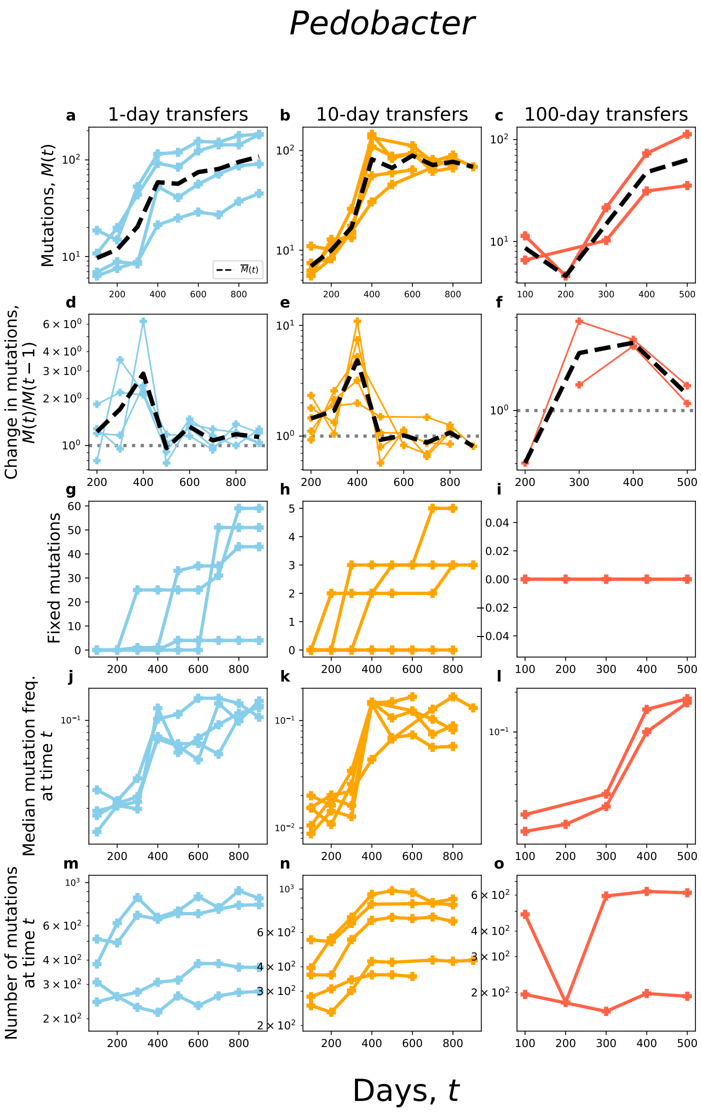

Figure S11: Molecular evolutionary dynamics of *Pedobacterium* sp. KBS0701. **a-c)** Cumulative mutation ($M(t)$) trajectories for both strains over time. The dashed black line is the mean. **d-f)** The change in $M(t)$ between timepoints across treatments. **g-i)** The cumulative number of fixed mutations over time for all populations within a given treatment. **j-l)** The median mutation frequency over time. **m-o)** The total number of polymorphic mutations segregating in the population over time.

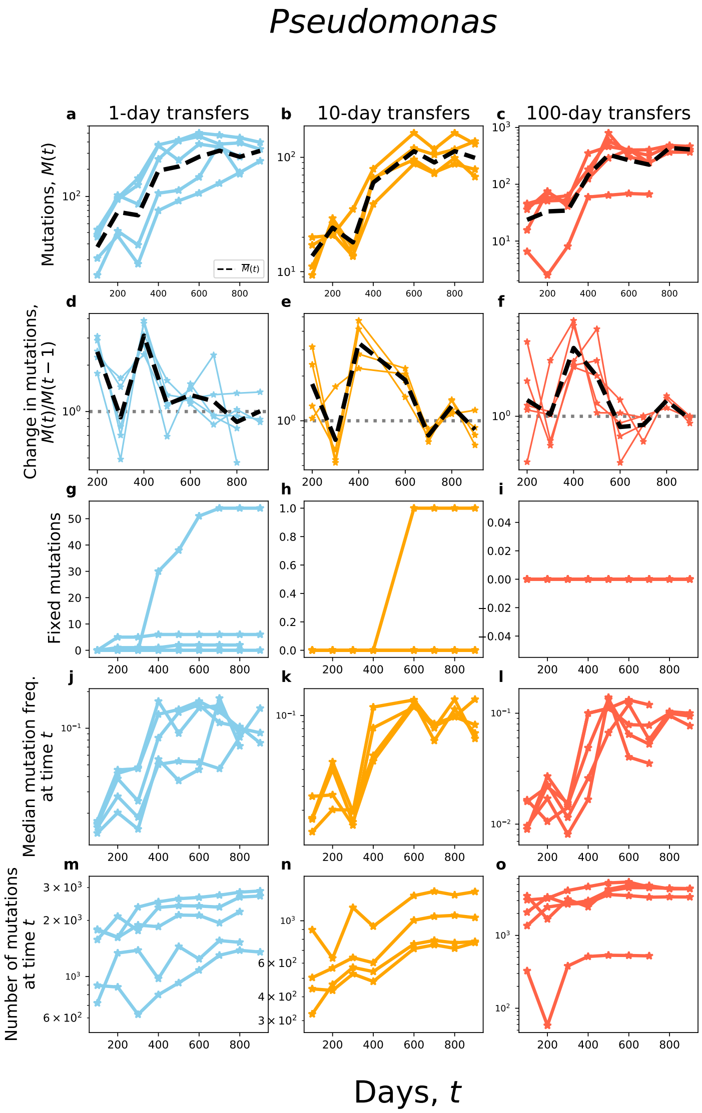

Figure S12: Molecular evolutionary dynamics of *Pseudomonas* sp. KBS0710. **a-c)** Cumulative mutation ($M(t)$) trajectories for both strains over time. The dashed black line is the mean. **d-f)** The change in $M(t)$ between timepoints across treatments. **g-i)** The cumulative number of fixed mutations over time for all populations within a given treatment. **j-l)** The median mutation frequency over time. **m-o)** The total number of polymorphic mutations segregating in the population over time.

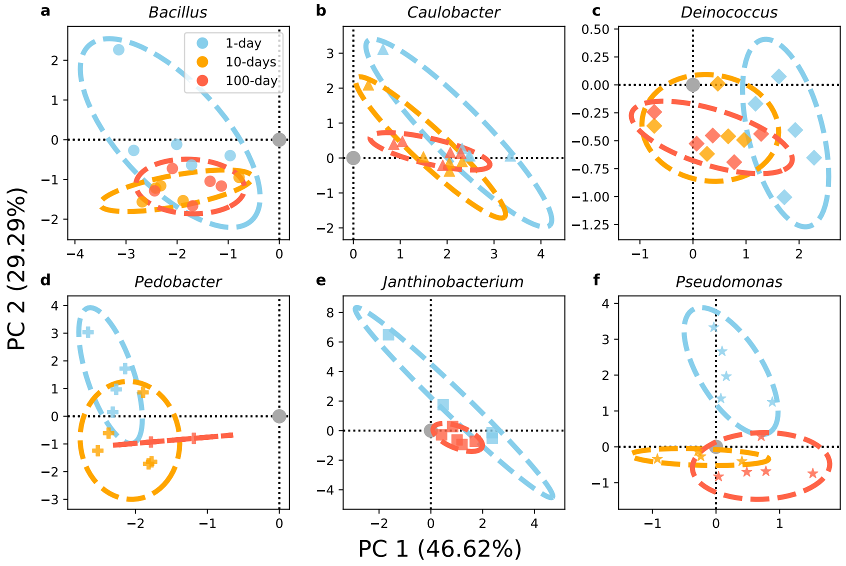

Figure S13: **a-f)** A single PCA was performed on the observed mutation spectra for all replicate populations, where each taxon has been plotted separately with 95% confidence ellipses for each treatment. Individual taxa are plotted as separate sub-plots for visual clarity.

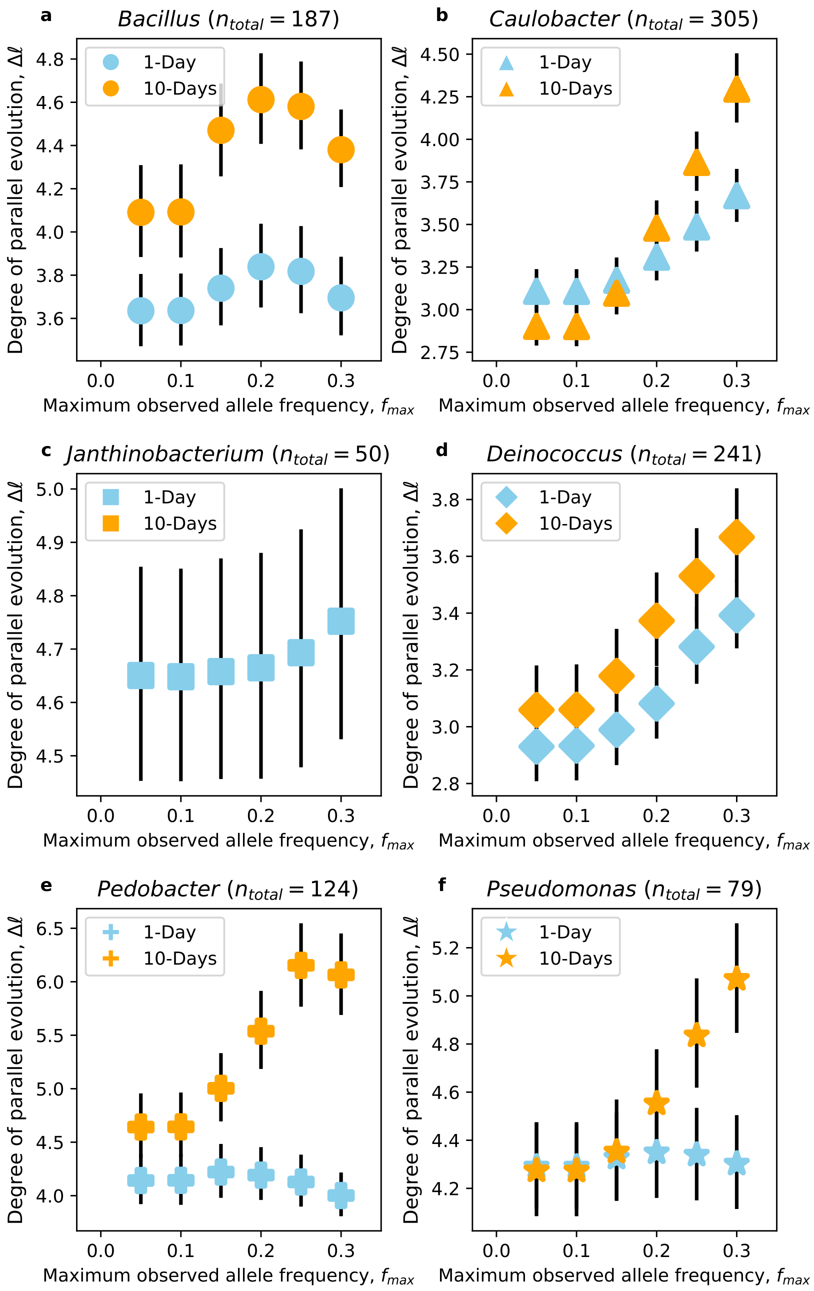

Figure S14: **a-f) a-f)** The relationship between the maximum observed frequency of a mutation (f_{max}) and the degree of parallelism. Genome-wide parallelism is generally higher among mutations that reach a higher maximum frequency during their sojourn time in the population. Dots and bars represent the mean and 95% CIs from 10,000 subsamples of mutations with a given f_{max} cutoff, respectively.

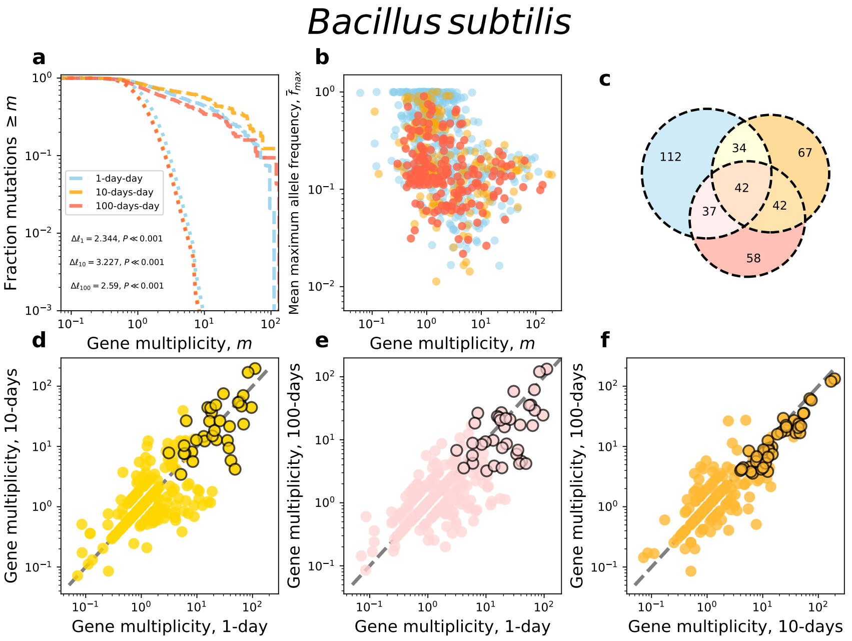

Figure S15: Parallelism and divergence/convergence visualizations for all mutations in *Bacillus*. **a**) The survival curve for multiplicity decays at a slower rate than the null across treatments, indicating that we can reject the null hypothesis that nonsynonymous mutations are equally distributed across genes. Dashed lines represent the empirical survival curves and dotted lines represent the null survival curves. The color scheme in **a** is used for all sub-plots. **b**) A scatterplot visualizing the relationship between the mean maximum frequency ($\overline{f}_{max}$) and multiplicity of genes. **c**) A venn diagram showing the overlap in genes that were significantly enriched for nonsynonymous mutations across treatments. **d-f**) Pairwise comparisons of multiplicity for genes across all treatments, where significantly enriched genes have a black outline.

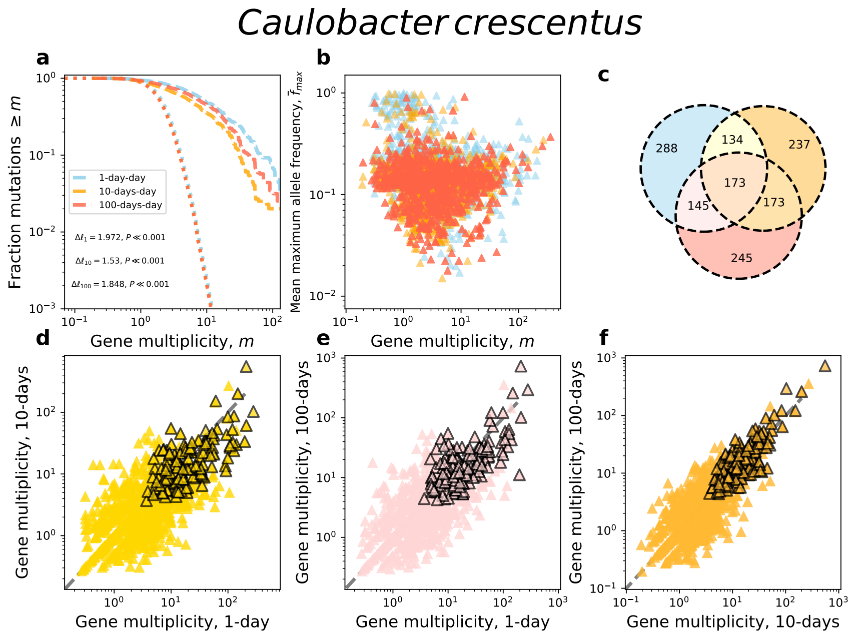

Figure S16: Parallelism and divergence/convergence analyses for all mutations in *Caulobacter*. **a**) The survival curve for multiplicity decays at a slower rate than the null across treatments, indicating that we can reject the null hypothesis that nonsynonymous mutations are equally distributed across genes. Dashed lines represent the empirical survival curves and dotted lines represent the null survival curves. The color scheme in **a** is used for all sub-plots. **b**) A scatterplot visualizing the relationship between the mean maximum frequency ($\overline{f}_{max}$) and multiplicity of genes. **c**) A venn diagram showing the overlap in genes that were significantly enriched for nonsynonymous mutations across treatments. **d-f**) Pairwise comparisons of multiplicity for genes across all treatments, where significantly enriched genes have a black outline.

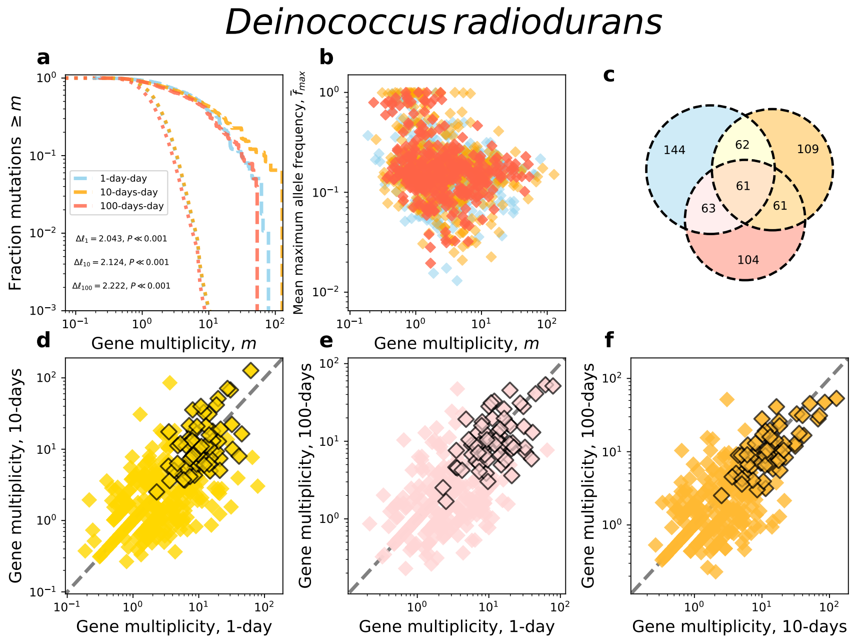

Figure S17: Parallelism and divergence/convergence analyses for all mutations in *Deinococcus*. **a**) The survival curve for multiplicity decays at a slower rate than the null across treatments, indicating that we can reject the null hypothesis that nonsynonymous mutations are equally distributed across genes. Dashed lines represent the empirical survival curves and dotted lines represent the null survival curves. The color scheme in **a** is used for all sub-plots. **b**) A scatterplot visualizing the relationship between the mean maximum frequency ($\overline{f}_{max}$) and multiplicity of genes. **c**) A venn diagram showing the overlap in genes that were significantly enriched for nonsynonymous mutations across treatments. **d-f**) Pairwise comparisons of multiplicity for genes across all treatments, where significantly enriched genes have a black outline.

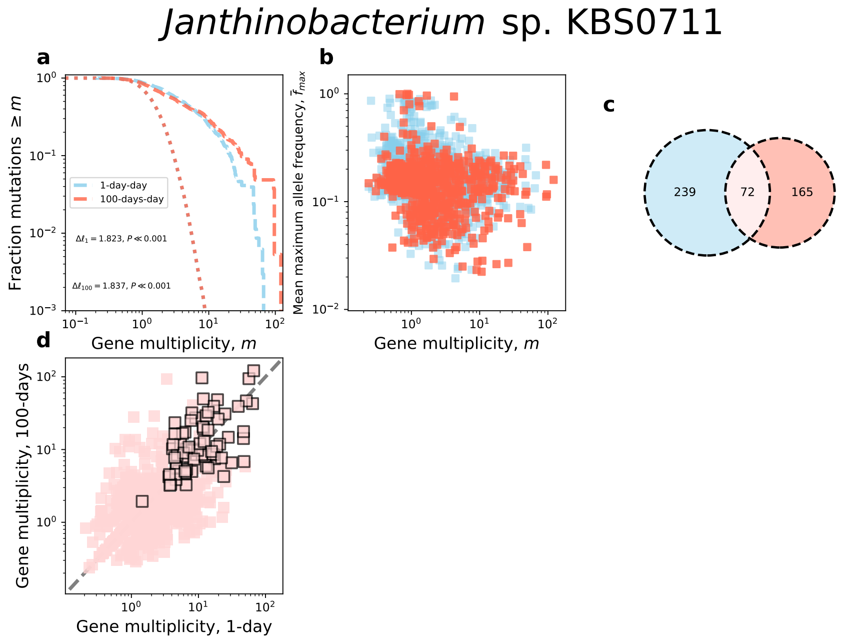

Figure S18: Parallelism and divergence/convergence analyses for all mutations in *Janthinobacterium*. **a**) The survival curve for multiplicity decays at a slower rate than the null across treatments, indicating that we can reject the null hypothesis that nonsynonymous mutations are equally distributed across genes. Dashed lines represent the empirical survival curves and dotted lines represent the null survival curves. The color scheme in **a** is used for all sub-plots. **b**) A scatterplot visualizing the relationship between the mean maximum frequency ($\overline{f}_{max}$) and multiplicity of genes. **c**) A venn diagram showing the overlap in genes that were significantly enriched for nonsynonymous mutations across treatments. **d-f**) Pairwise comparisons of multiplicity for genes across all treatments, where significantly enriched genes have a black outline.

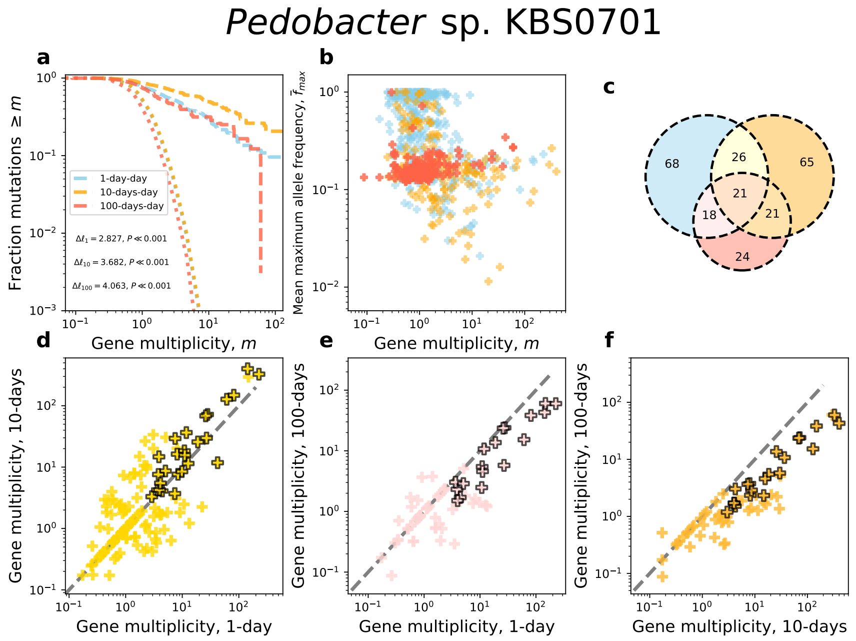

Figure S19: Parallelism and divergence/convergence analyses for all mutations in *Pedobacter*. **a**) The survival curve for multiplicity decays at a slower rate than the null across treatments, indicating that we can reject the null hypothesis that nonsynonymous mutations are equally distributed across genes. Dashed lines represent the empirical survival curves and dotted lines represent the null survival curves. The color scheme in **a** is used for all sub-plots. **b**) A scatterplot visualizing the relationship between the mean maximum frequency ($\overline{f}_{max}$) and multiplicity of genes. **c**) A venn diagram showing the overlap in genes that were significantly enriched for nonsynonymous mutations across treatments. **d-f**) Pairwise comparisons of multiplicity for genes across all treatments, where significantly enriched genes have a black outline.

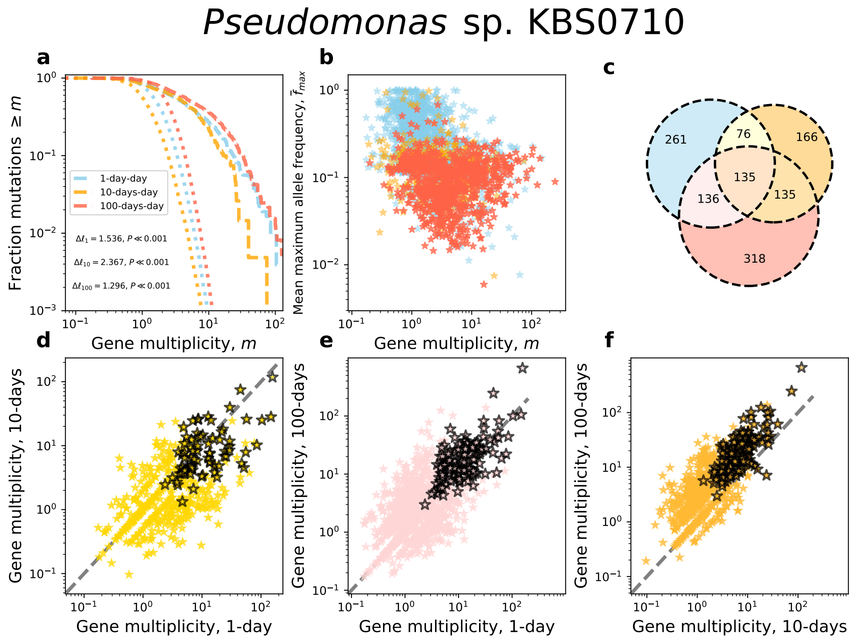

Figure S20: Parallelism and divergence/convergence analyses for all mutations in *Pseudomonas*. **a**) The survival curve for multiplicity decays at a slower rate than the null across treatments, indicating that we can reject the null hypothesis that nonsynonymous mutations are equally distributed across genes. Dashed lines represent the empirical survival curves and dotted lines represent the null survival curves. The color scheme in **a** is used for all sub-plots. **b**) A scatterplot visualizing the relationship between the mean maximum frequency ($\overline{f}_{max}$) and multiplicity of genes. **c**) A venn diagram showing the overlap in genes that were significantly enriched for nonsynonymous mutations across treatments. **d-f**) Pairwise comparisons of multiplicity for genes across all treatments, where significantly enriched genes have a black outline.

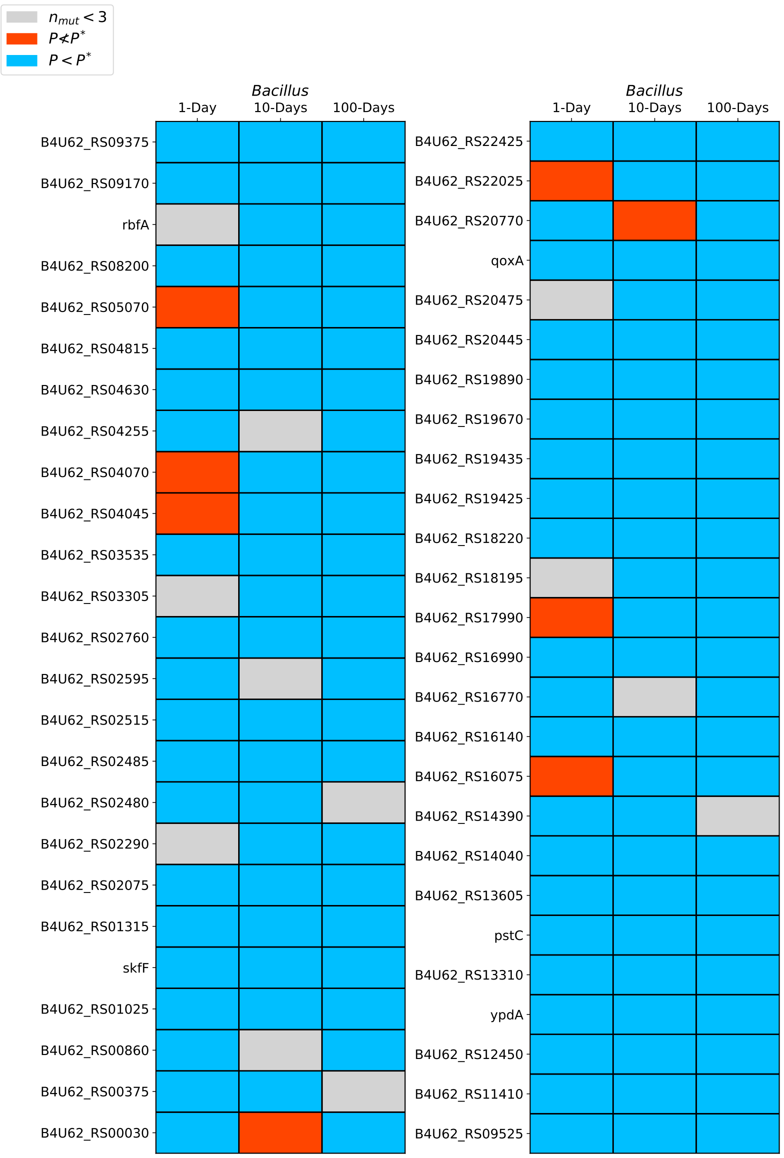

Figure S21: Visualization of all genes with an excess of non-synonymous mutations in more than one treatment for *Bacillus*. If a gene acquired more than three mutations ($n_{min} \geq 3$) we tested whether it acquired more mutations than expected by chance. If the P-value of a given gene was less than the FDR-corrected P-values ($P < P^*$) it was significantly enriched for mutations, otherwise it was not significantly enriched (PP^*).

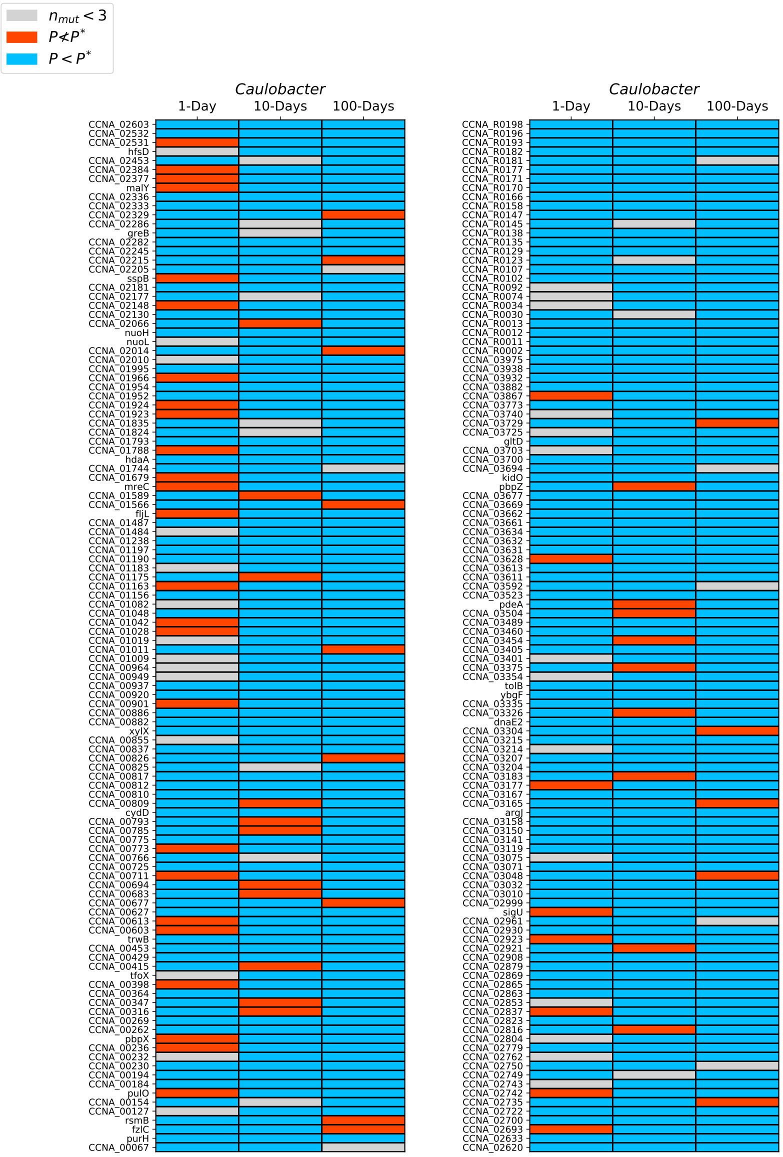

Figure S22: Visualization of all genes with an excess of non-synonymous mutations in more than one treatment for *Caulobacter*. If a gene acquired more than three mutations ($n_{min} \geq 3$) we tested whether it acquired more mutations than expected by chance. If the P-value of a given gene was less than the FDR-corrected P-values ($P < P^*$) it was significantly enriched for mutations, otherwise it was not significantly enriched (PP^*).

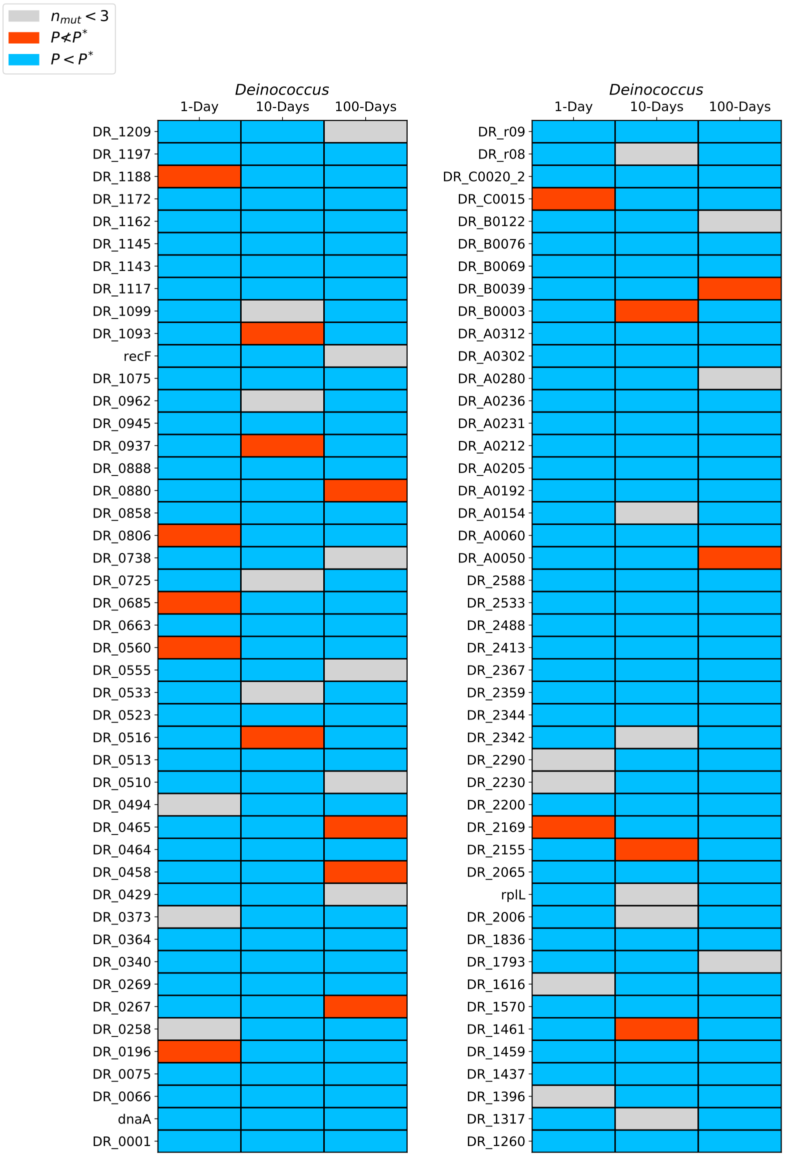

Figure S23: Visualization of all genes with an excess of non-synonymous mutations in more than one treatment for *Deinococcus*. If a gene acquired more than three mutations ($n_{min} \geq 3$) we tested whether it acquired more mutations than expected by chance. If the P-value of a given gene was less than the FDR-corrected P-values ($P < P^*$) it was significantly enriched for mutations, otherwise it was not significantly enriched (PP^*).

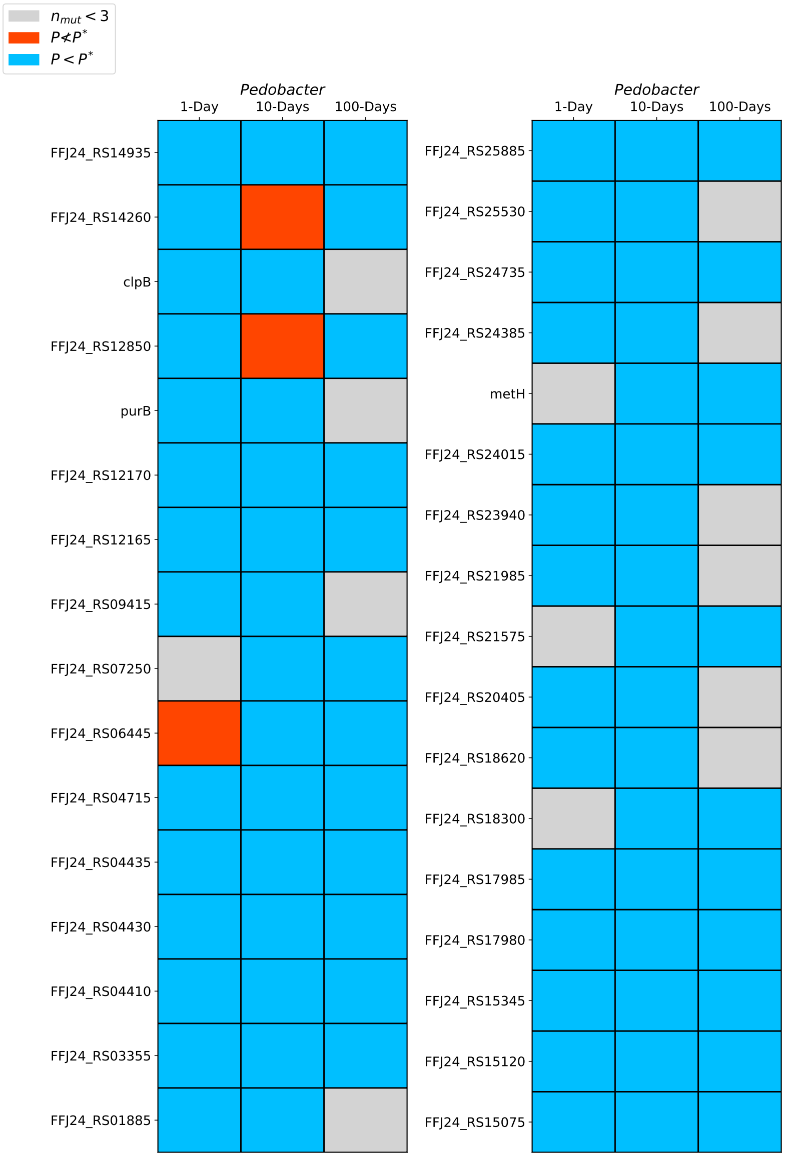

Figure S24: Visualization of all genes with an excess of non-synonymous mutations in more than one treatment for *Pedobacter*. If a gene acquired more than three mutations ($n_{min} \geq 3$) we tested whether it acquired more mutations than expected by chance. If the P-value of a given gene was less than the FDR-corrected P-values ($P < P^*$) it was significantly enriched for mutations, otherwise it was not significantly enriched (PP^*).

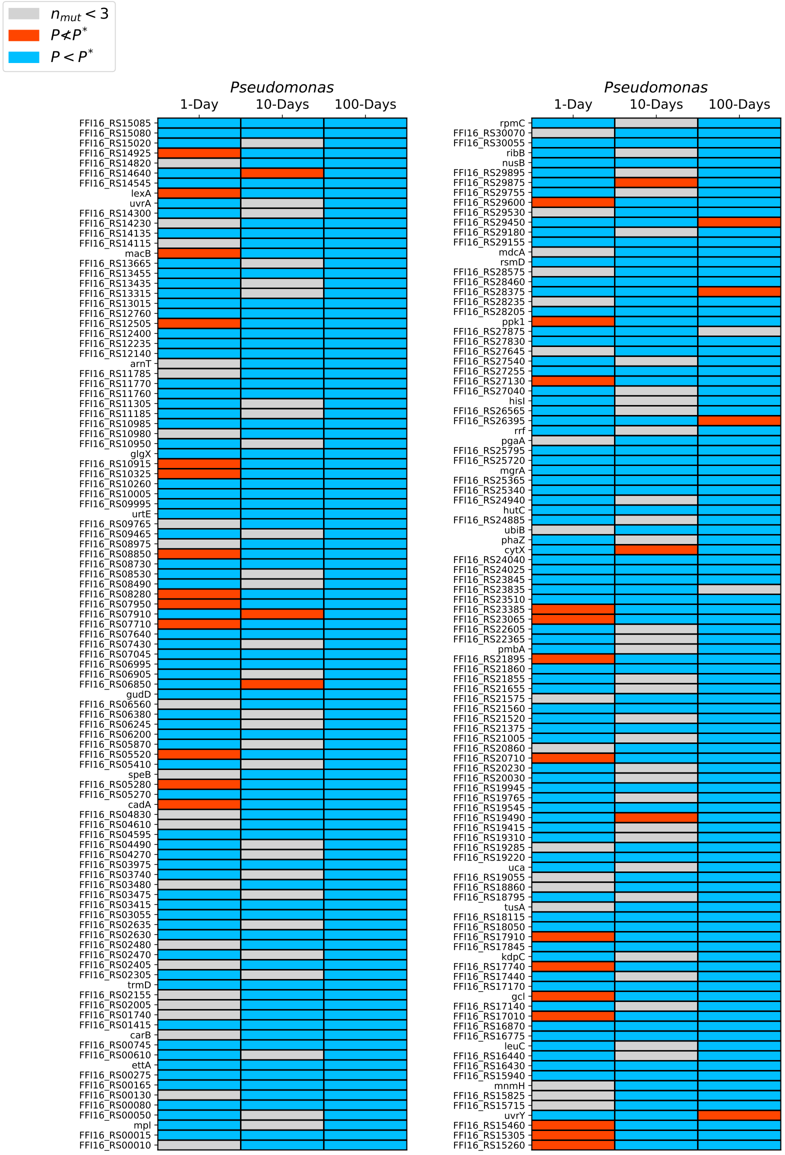

Figure S25: Visualization of all genes with an excess of non-synonymous mutations in more than one treatment for *Pseudomonas*. If a gene acquired more than three mutations ($n_{min} \geq 3$) we tested whether it acquired more mutations than expected by chance. If the P-value of a given gene was less than the FDR-corrected P-values ($P < P^*$) it was significantly enriched for mutations, otherwise it was not significantly enriched (PP^*).
