# Supplementary material for: Molecular Evolutionary Dynamics of Energy Limited Microorganisms
Source: Mol Biol Evol. 2021 Jul 13;38(10):4532–45. doi: 10.1093/molbev/msab195 (PMC8476154; doi:10.1093/molbev/msab195)
Supplement: msab195_Supplementary_Data [file msab195_supplementary_data.zip › supplement_latex.zip!/supplement_latex/figures/multiplicity_B.pdf]

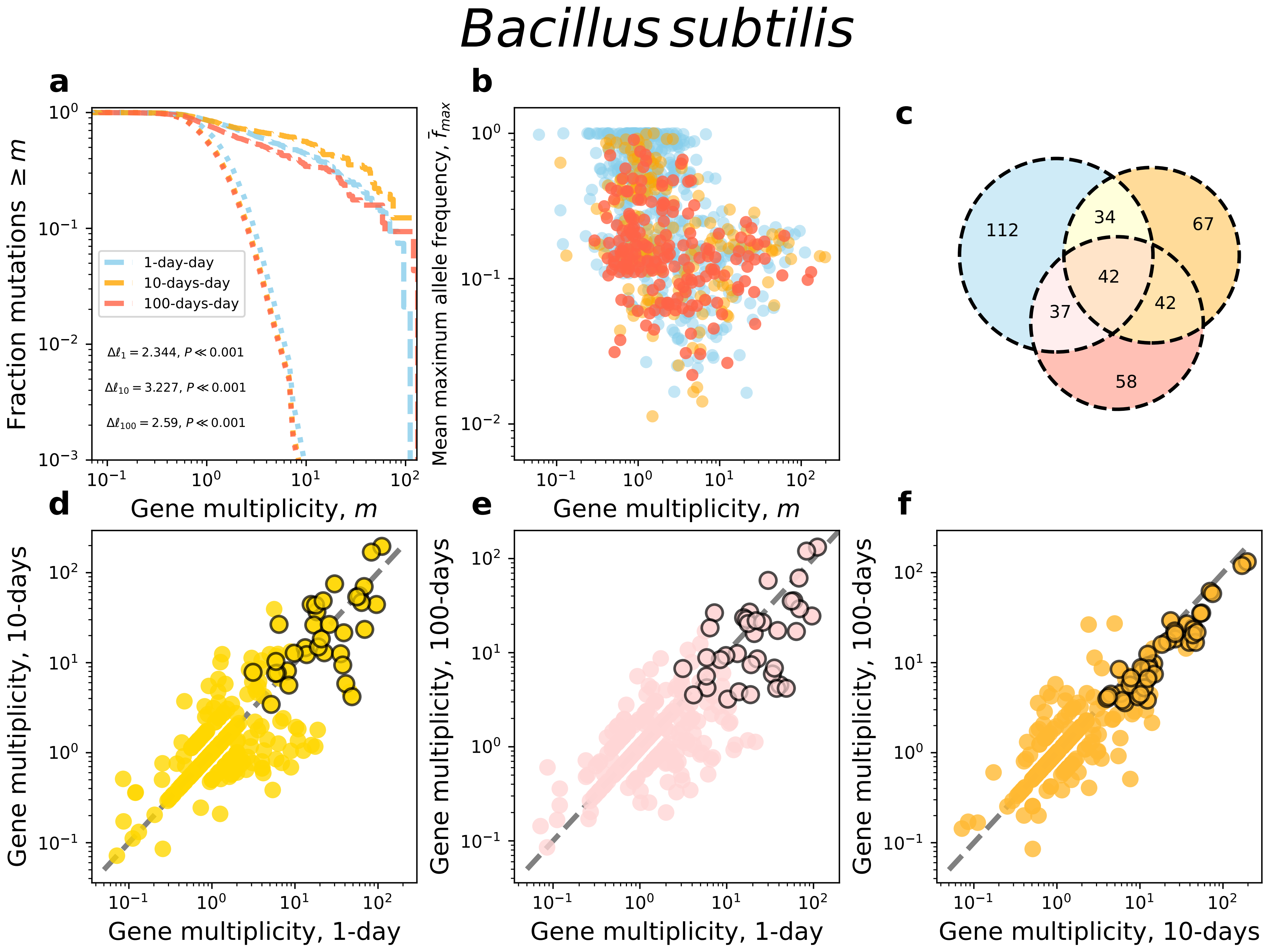

Bacillus subtilis
a
b
c
d
e
f
Fraction mutations ≥ m
1-day-day
10-days-day
100-days-day
$\Delta\ell_1 = 2.344, P \ll 0.001$
$\Delta\ell_{10} = 3.227, P \ll 0.001$
$\Delta\ell_{100} = 2.59, P \ll 0.001$
Gene multiplicity, m
Mean maximum allele frequency, $\bar{f}_{max}$
112
34
67
42
37
42
58
Gene multiplicity, 10-days
Gene multiplicity, 1-day
Gene multiplicity, 100-days
Gene multiplicity, 1-day
Gene multiplicity, 100-days
Gene multiplicity, 10-days
