# Supplementary material for: Molecular Evolutionary Dynamics of Energy Limited Microorganisms
Source: Mol Biol Evol. 2021 Jul 13;38(10):4532–45. doi: 10.1093/molbev/msab195 (PMC8476154; doi:10.1093/molbev/msab195)
Supplement: msab195_Supplementary_Data [file msab195_supplementary_data.zip › supplement_latex.zip!/supplement_latex/figures/multiplicity_C.pdf]

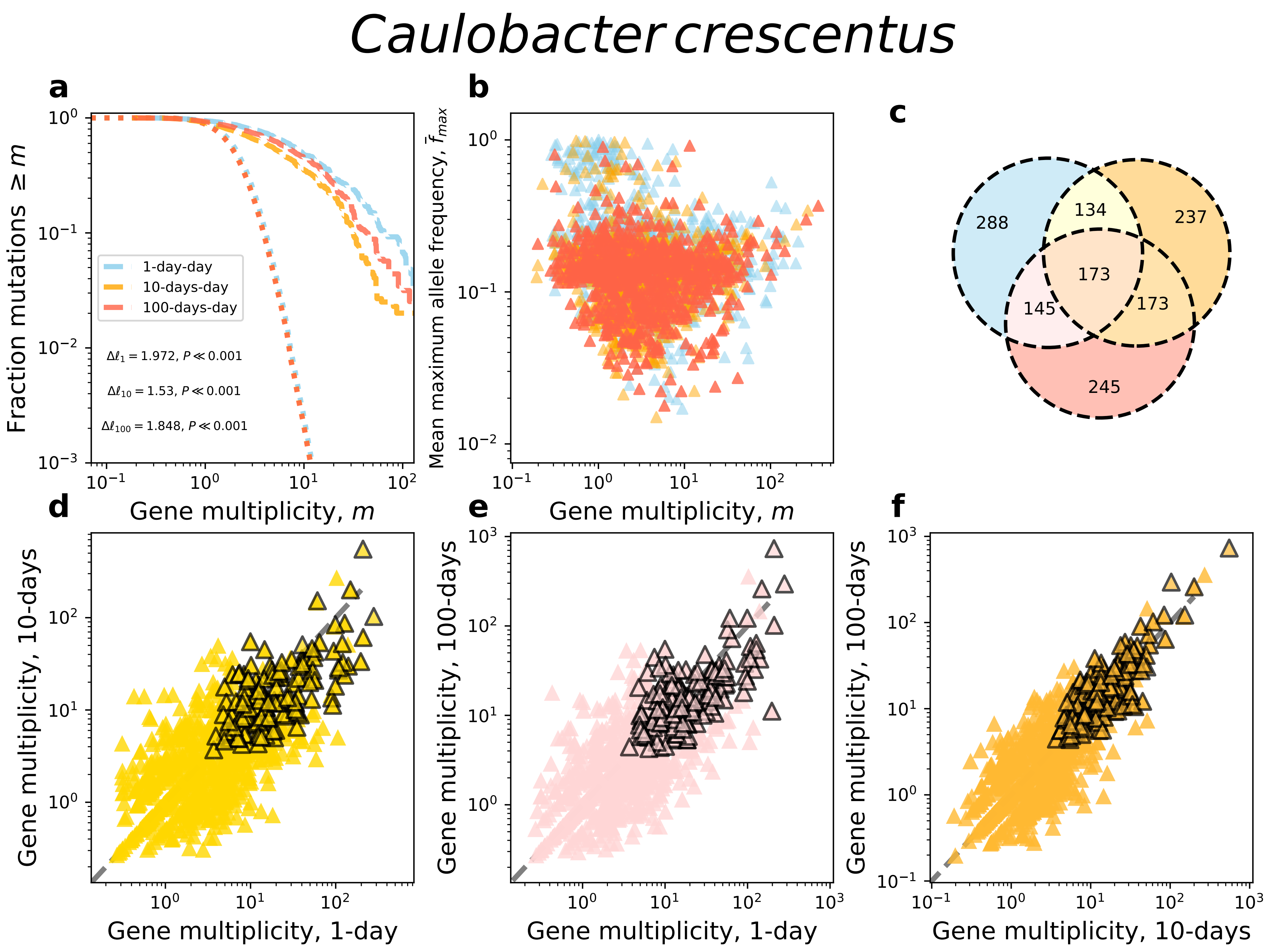

Caulobacter crescentus
a
b
c
d
e
f
Fraction mutations ≥ m
Gene multiplicity, m
1-day-day
10-days-day
100-days-day
Δℓ1 = 1.972, P ≪ 0.001
Δℓ10 = 1.53, P ≪ 0.001
Δℓ100 = 1.848, P ≪ 0.001
Mean maximum allele frequency, f̄max
288
134
237
173
145
173
245
Gene multiplicity, 10-days
Gene multiplicity, 1-day
Gene multiplicity, 100-days
Gene multiplicity, 10-days
