# Supplementary material for: Molecular Evolutionary Dynamics of Energy Limited Microorganisms
Source: Mol Biol Evol. 2021 Jul 13;38(10):4532–45. doi: 10.1093/molbev/msab195 (PMC8476154; doi:10.1093/molbev/msab195)
Supplement: msab195_Supplementary_Data [file msab195_supplementary_data.zip › supplement_latex.zip!/supplement_latex/figures/rate_C.pdf]

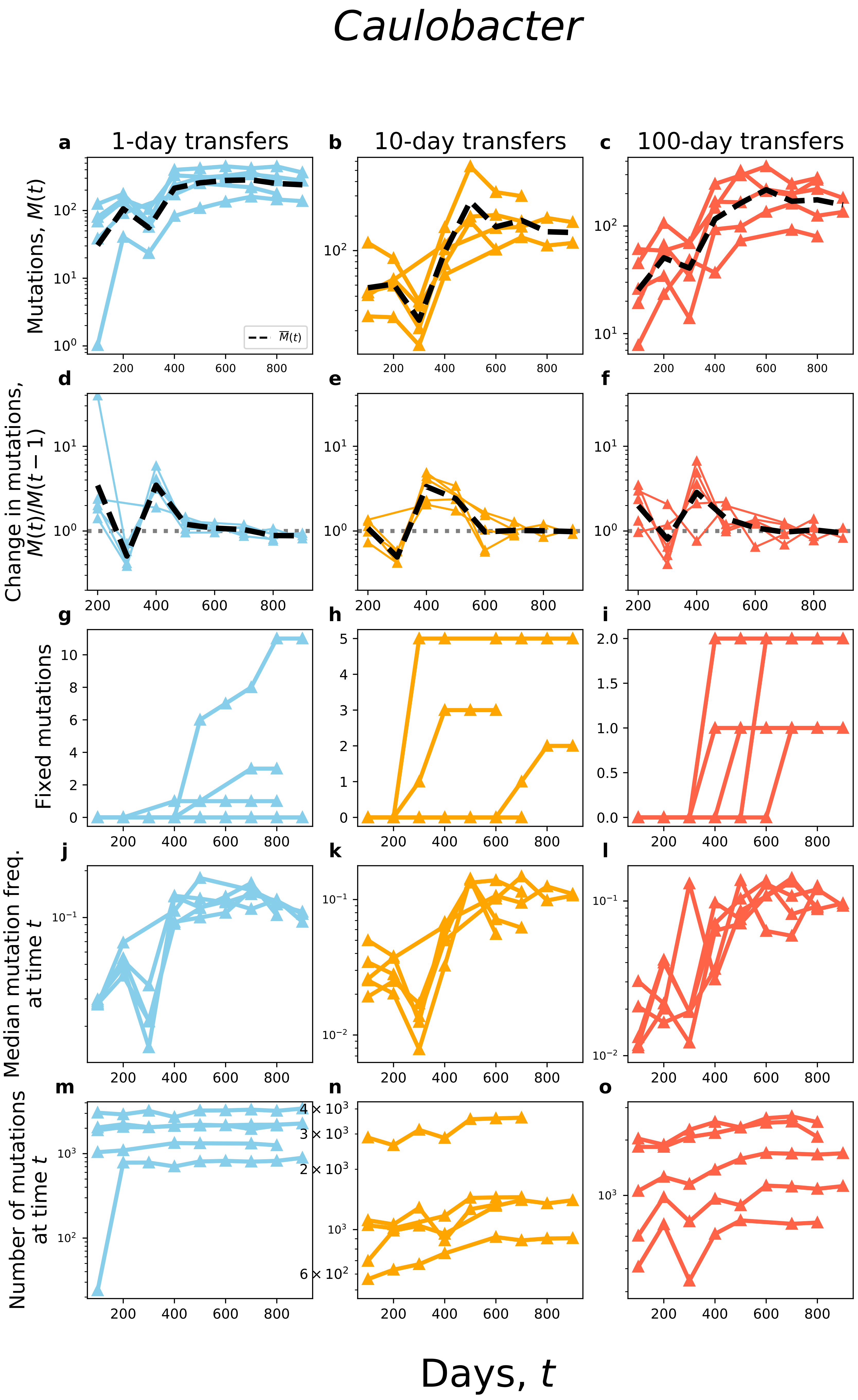

Caulobacter
a
1-day transfers
b
10-day transfers
c
100-day transfers
Mutations, M(t)
M̄(t)
d
e
f
Change in mutations, M(t)/M(t−1)
g
h
i
Fixed mutations
j
k
l
Median mutation freq. at time t
m
n
o
Number of mutations at time t
Days, t
