# Supplementary material for: Molecular Evolutionary Dynamics of Energy Limited Microorganisms
Source: Mol Biol Evol. 2021 Jul 13;38(10):4532–45. doi: 10.1093/molbev/msab195 (PMC8476154; doi:10.1093/molbev/msab195)
Supplement: msab195_Supplementary_Data [file msab195_supplementary_data.zip › supplement_latex.zip!/supplement_latex/figures/G_score_vs_fmax_subsample.pdf]

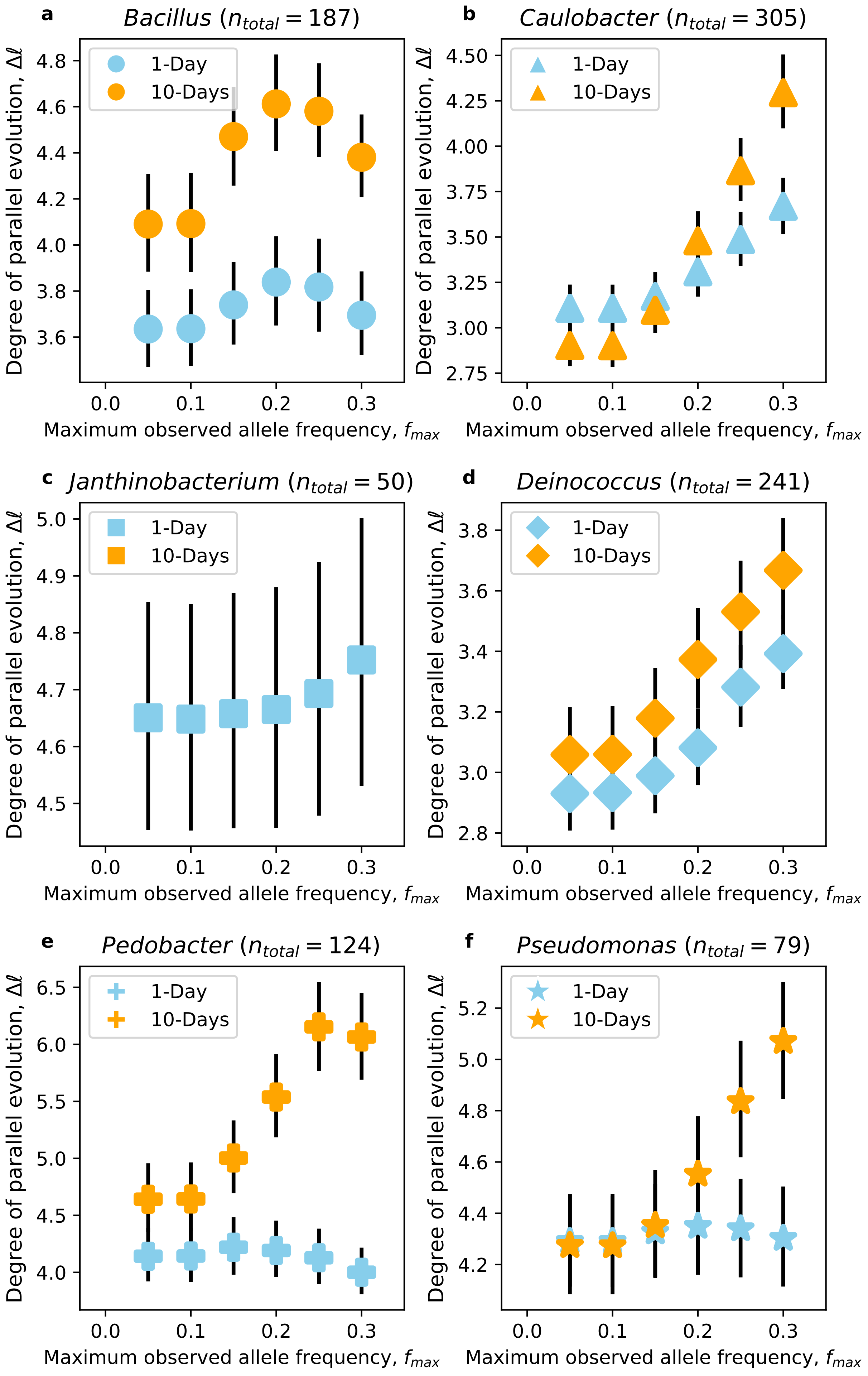

a
Bacillus ($n_{total} = 187$)
1-Day
10-Days
Degree of parallel evolution, $\Delta\ell$
Maximum observed allele frequency, f_{max}
b
Caulobacter ($n_{total} = 305$)
1-Day
10-Days
Degree of parallel evolution, $\Delta\ell$
Maximum observed allele frequency, f_{max}
c
Janthinobacterium ($n_{total} = 50$)
1-Day
10-Days
Degree of parallel evolution, $\Delta\ell$
Maximum observed allele frequency, f_{max}
d
Deinococcus ($n_{total} = 241$)
1-Day
10-Days
Degree of parallel evolution, $\Delta\ell$
Maximum observed allele frequency, f_{max}
e
Pedobacter ($n_{total} = 124$)
1-Day
10-Days
Degree of parallel evolution, $\Delta\ell$
Maximum observed allele frequency, f_{max}
f
Pseudomonas ($n_{total} = 79$)
1-Day
10-Days
Degree of parallel evolution, $\Delta\ell$
Maximum observed allele frequency, f_{max}
