# Supplementary material for: Molecular Evolutionary Dynamics of Energy Limited Microorganisms
Source: Mol Biol Evol. 2021 Jul 13;38(10):4532–45. doi: 10.1093/molbev/msab195 (PMC8476154; doi:10.1093/molbev/msab195)
Supplement: msab195_Supplementary_Data [file msab195_supplementary_data.zip › supplement_latex.zip!/supplement_latex/figures/mutation_spectra_pca.pdf]

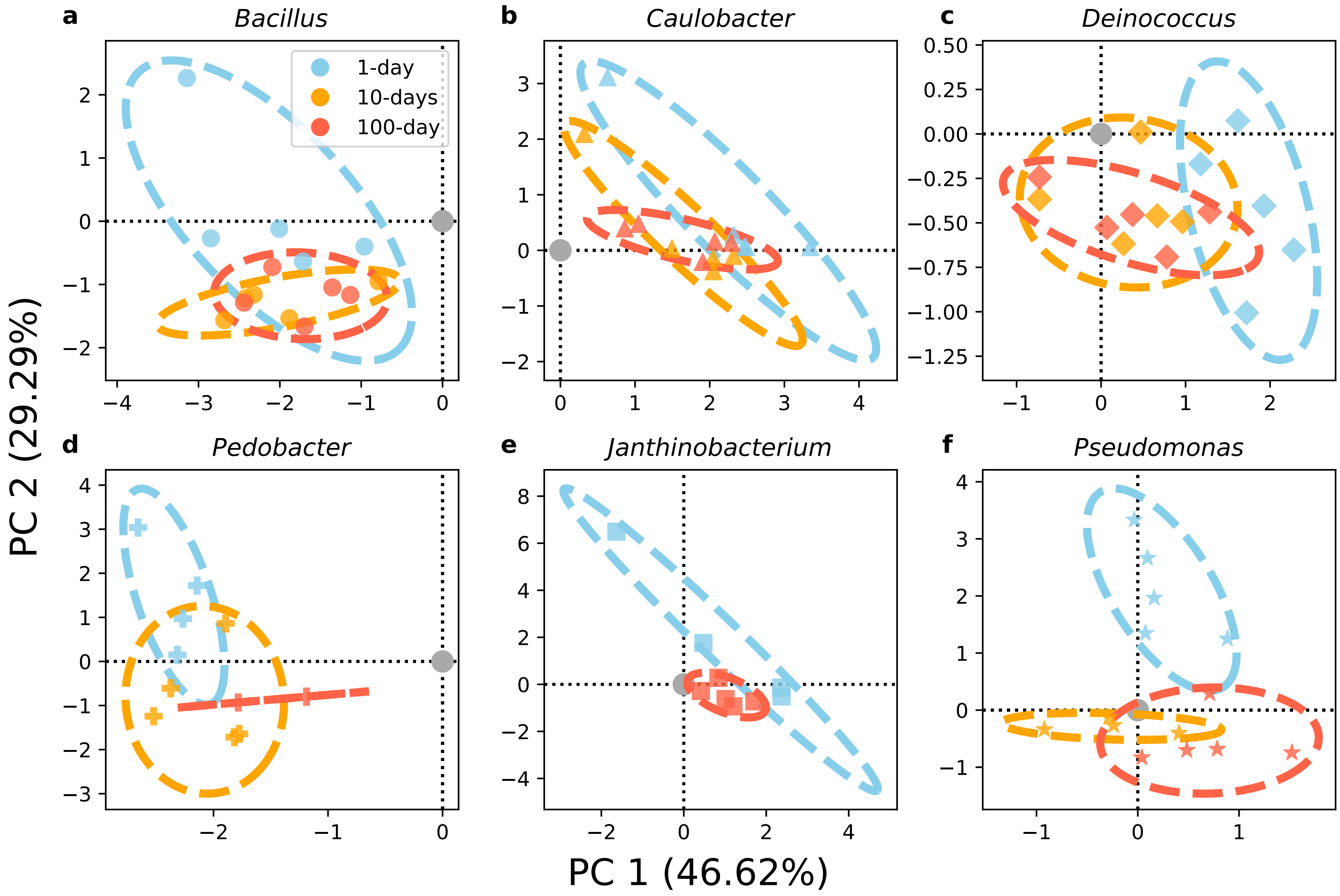

a
Bacillus
1-day
10-days
100-day
b
Caulobacter
c
Deinococcus
d
Pedobacter
e
Janthinobacterium
f
Pseudomonas
PC 2 (29.29%)
PC 1 (46.62%)
