# Supplementary material for: Molecular Evolutionary Dynamics of Energy Limited Microorganisms
Source: Mol Biol Evol. 2021 Jul 13;38(10):4532–45. doi: 10.1093/molbev/msab195 (PMC8476154; doi:10.1093/molbev/msab195)
Supplement: msab195_Supplementary_Data [file msab195_supplementary_data.zip › supplement_latex.zip!/supplement_latex/figures/gene_convergence_plot_C.pdf]

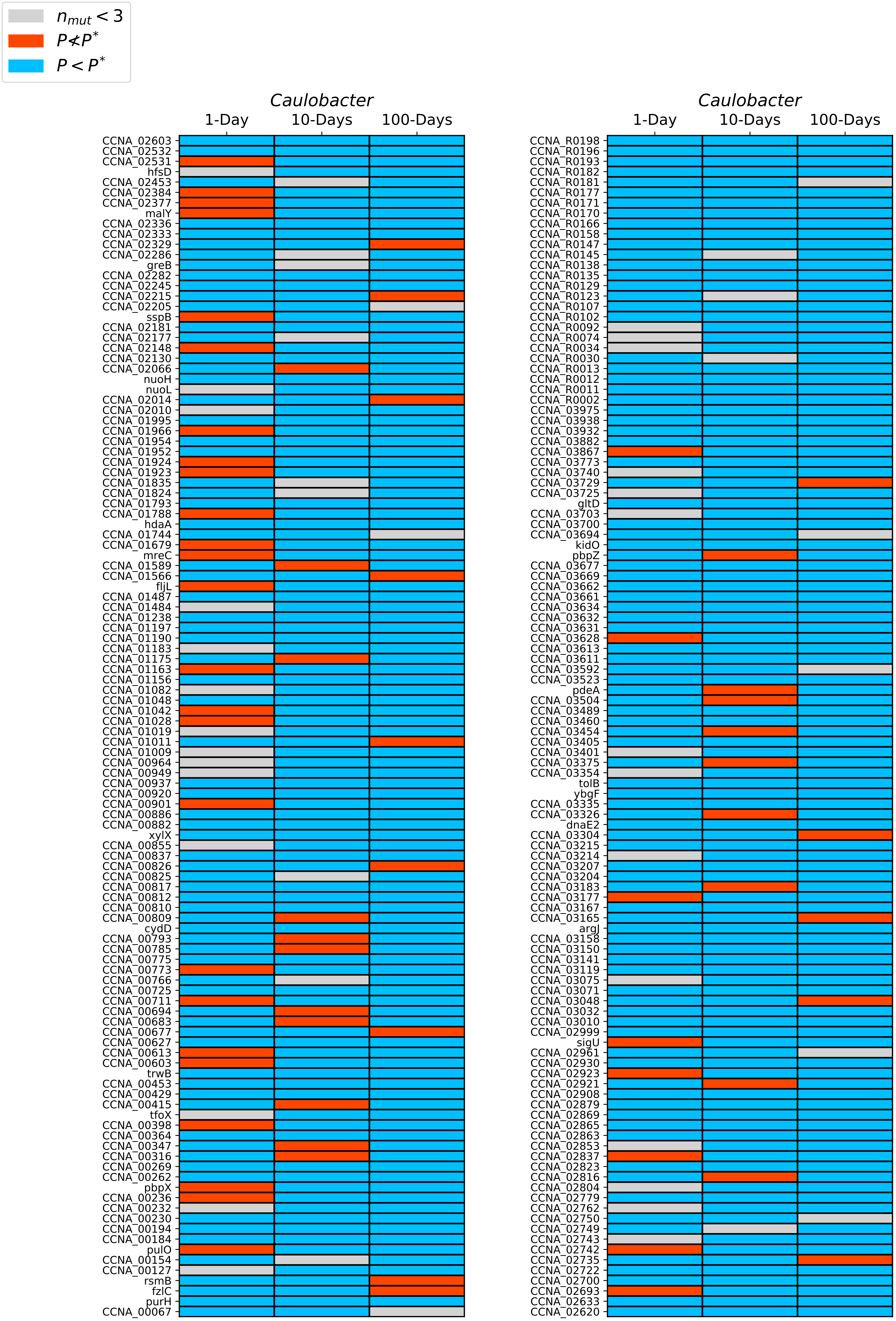

$n_{mut} < 3$
$P \nless P^*$
$P < P^*$
Caulobacter
1-Day
10-Days
100-Days
CCNA_02603
CCNA_02532
CCNA_02531
hfsD
CCNA_02453
CCNA_02384
CCNA_02377
malY
CCNA_02336
CCNA_02333
CCNA_02329
CCNA_02286
greB
CCNA_02282
CCNA_02245
CCNA_02215
CCNA_02205
sspB
CCNA_02181
CCNA_02177
CCNA_02148
CCNA_02130
CCNA_02066
nuoH
nuoL
CCNA_02014
CCNA_02010
CCNA_01995
CCNA_01966
CCNA_01954
CCNA_01952
CCNA_01924
CCNA_01923
CCNA_01835
CCNA_01824
CCNA_01793
CCNA_01788
hdaA
CCNA_01744
CCNA_01679
mreC
CCNA_01589
CCNA_01566
fljL
CCNA_01487
CCNA_01484
CCNA_01238
CCNA_01197
CCNA_01190
CCNA_01183
CCNA_01175
CCNA_01163
CCNA_01156
CCNA_01082
CCNA_01048
CCNA_01042
CCNA_01028
CCNA_01019
CCNA_01011
CCNA_01009
CCNA_00964
CCNA_00949
CCNA_00937
CCNA_00920
CCNA_00901
CCNA_00886
CCNA_00882
xylX
CCNA_00855
CCNA_00837
CCNA_00826
CCNA_00825
CCNA_00817
CCNA_00812
CCNA_00810
CCNA_00809
cydD
CCNA_00793
CCNA_00785
CCNA_00775
CCNA_00773
CCNA_00766
CCNA_00725
CCNA_00711
CCNA_00694
CCNA_00683
CCNA_00677
CCNA_00627
CCNA_00613
CCNA_00603
trwB
CCNA_00453
CCNA_00429
CCNA_00415
tfoX
CCNA_00398
CCNA_00364
CCNA_00347
CCNA_00316
CCNA_00269
CCNA_00262
pbpX
CCNA_00236
CCNA_00232
CCNA_00230
CCNA_00194
CCNA_00184
pulO
CCNA_00154
CCNA_00127
rsmB
fzlC
purH
CCNA_00067
Caulobacter
1-Day
10-Days
100-Days
CCNA_R0198
CCNA_R0196
CCNA_R0193
CCNA_R0182
CCNA_R0181
CCNA_R0177
CCNA_R0171
CCNA_R0170
CCNA_R0166
CCNA_R0158
CCNA_R0147
CCNA_R0145
CCNA_R0138
CCNA_R0135
CCNA_R0129
CCNA_R0123
CCNA_R0107
CCNA_R0102
CCNA_R0092
CCNA_R0074
CCNA_R0034
CCNA_R0030
CCNA_R0013
CCNA_R0012
CCNA_R0011
CCNA_R0002
CCNA_03975
CCNA_03938
CCNA_03932
CCNA_03882
CCNA_03867
CCNA_03773
CCNA_03740
CCNA_03729
CCNA_03725
gltD
CCNA_03703
CCNA_03700
CCNA_03694
kidO
pbpZ
CCNA_03677
CCNA_03669
CCNA_03662
CCNA_03661
CCNA_03634
CCNA_03632
CCNA_03631
CCNA_03628
CCNA_03613
CCNA_03611
CCNA_03592
CCNA_03523
pdeA
CCNA_03504
CCNA_03489
CCNA_03460
CCNA_03454
CCNA_03405
CCNA_03401
CCNA_03375
CCNA_03354
tolB
ybgF
CCNA_03335
CCNA_03326
dnaE2
CCNA_03304
CCNA_03215
CCNA_03214
CCNA_03207
CCNA_03204
CCNA_03183
CCNA_03177
CCNA_03167
CCNA_03165
argJ
CCNA_03158
CCNA_03150
CCNA_03141
CCNA_03119
CCNA_03075
CCNA_03071
CCNA_03048
CCNA_03032
CCNA_03010
CCNA_02999
sigU
CCNA_02961
CCNA_02930
CCNA_02923
CCNA_02921
CCNA_02908
CCNA_02879
CCNA_02869
CCNA_02865
CCNA_02863
CCNA_02853
CCNA_02837
CCNA_02823
CCNA_02816
CCNA_02804
CCNA_02779
CCNA_02762
CCNA_02750
CCNA_02749
CCNA_02743
CCNA_02742
CCNA_02735
CCNA_02722
CCNA_02700
CCNA_02693
CCNA_02633
CCNA_02620
