# Supplementary material for: Molecular Evolutionary Dynamics of Energy Limited Microorganisms
Source: Mol Biol Evol. 2021 Jul 13;38(10):4532–45. doi: 10.1093/molbev/msab195 (PMC8476154; doi:10.1093/molbev/msab195)

Janthinobacterium sp. KBS0711

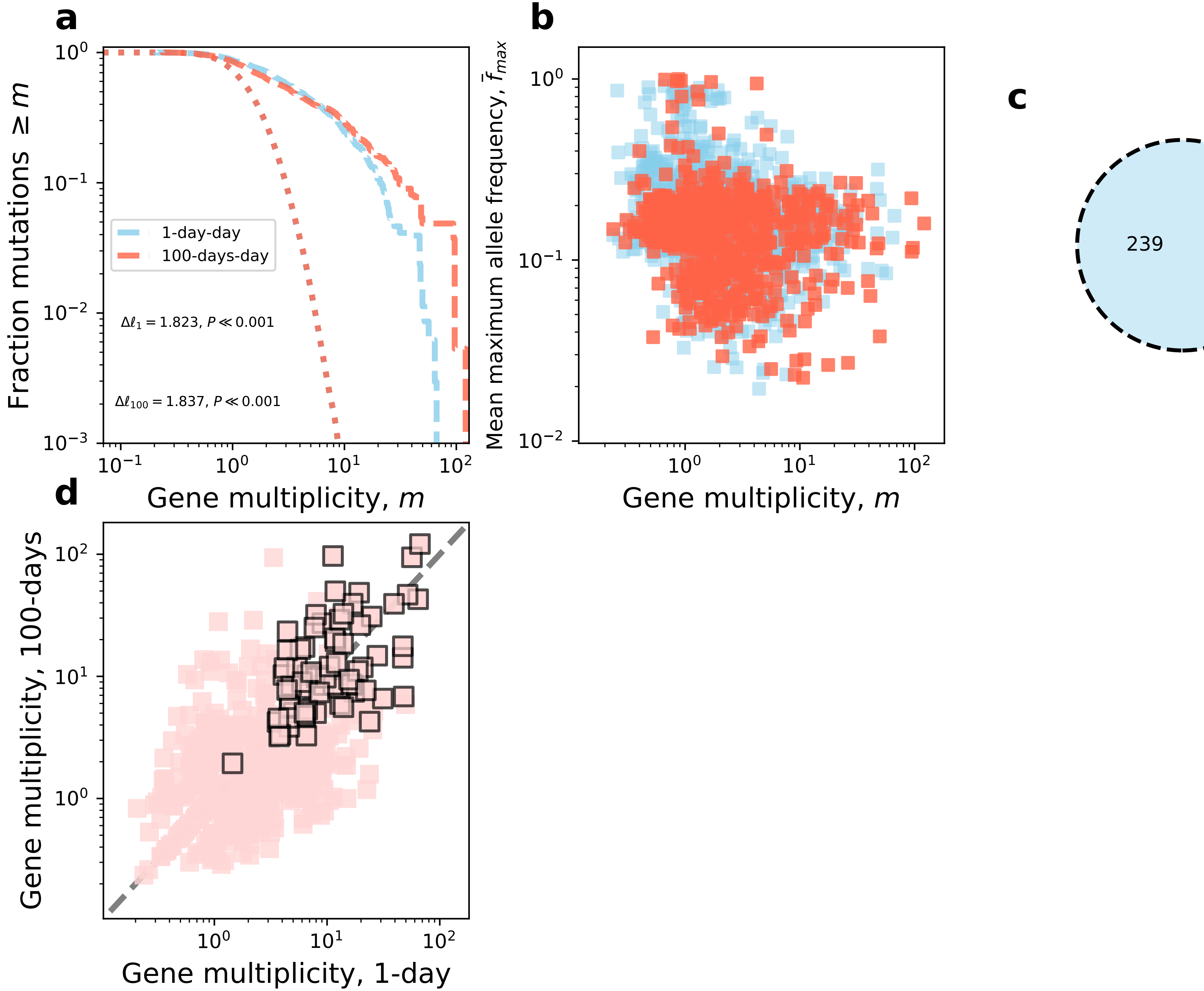

Supplement: msab195_Supplementary_Data [file msab195_supplementary_data.zip › supplement_latex.zip!/supplement_latex/figures/multiplicity_J.pdf]
