# Supplementary material for: Molecular Evolutionary Dynamics of Energy Limited Microorganisms
Source: Mol Biol Evol. 2021 Jul 13;38(10):4532–45. doi: 10.1093/molbev/msab195 (PMC8476154; doi:10.1093/molbev/msab195)
Supplement: msab195_Supplementary_Data [file msab195_supplementary_data.zip › supplement_latex.zip!/supplement_latex/figures/gene_convergence_plot_B.pdf]

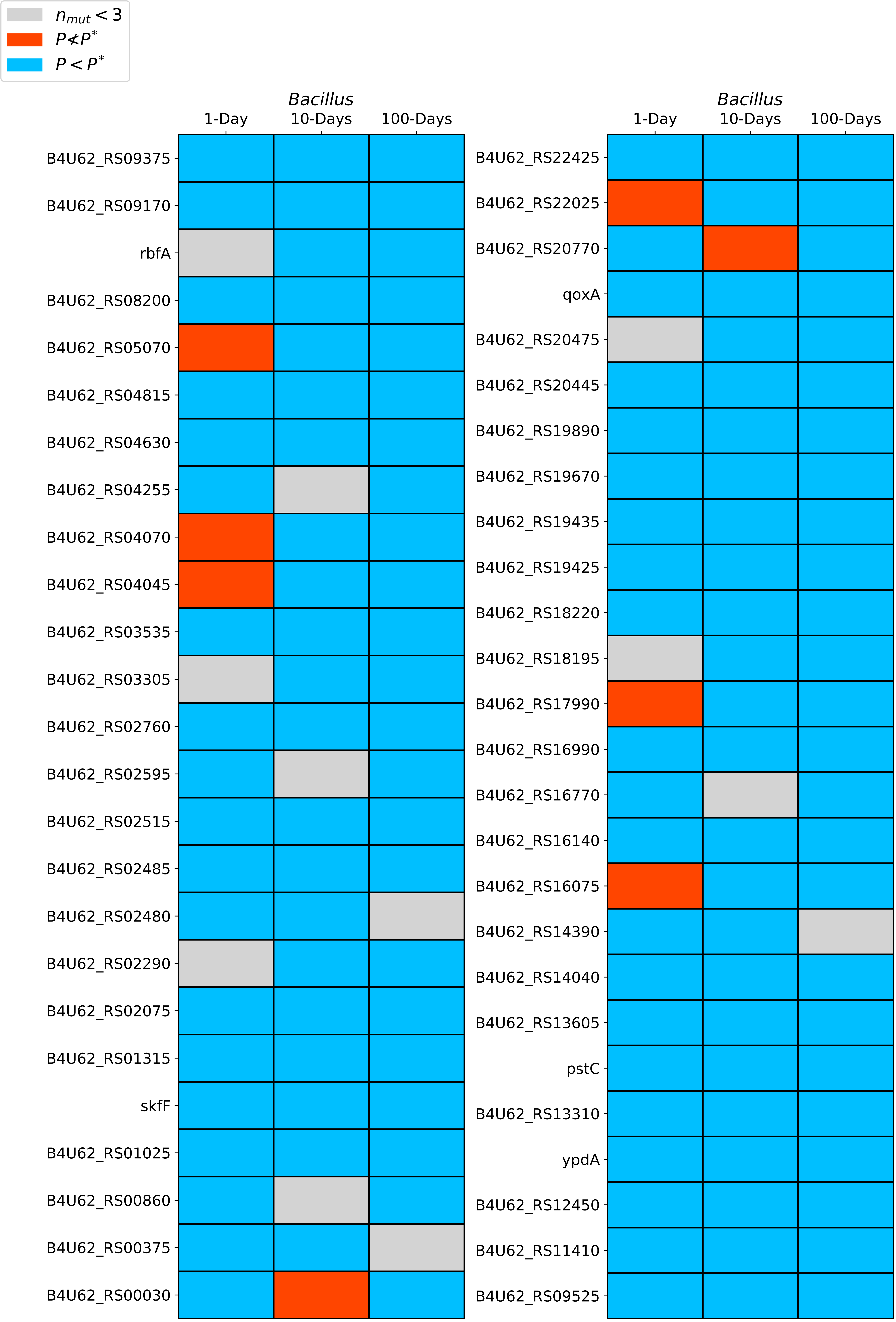

$n_{mut} < 3$
$P \not< P^*$
$P < P^*$
Bacillus
1-Day
10-Days
100-Days
B4U62_RS09375
B4U62_RS09170
rbfA
B4U62_RS08200
B4U62_RS05070
B4U62_RS04815
B4U62_RS04630
B4U62_RS04255
B4U62_RS04070
B4U62_RS04045
B4U62_RS03535
B4U62_RS03305
B4U62_RS02760
B4U62_RS02595
B4U62_RS02515
B4U62_RS02485
B4U62_RS02480
B4U62_RS02290
B4U62_RS02075
B4U62_RS01315
skfF
B4U62_RS01025
B4U62_RS00860
B4U62_RS00375
B4U62_RS00030
Bacillus
1-Day
10-Days
100-Days
B4U62_RS22425
B4U62_RS22025
B4U62_RS20770
qoxA
B4U62_RS20475
B4U62_RS20445
B4U62_RS19890
B4U62_RS19670
B4U62_RS19435
B4U62_RS19425
B4U62_RS18220
B4U62_RS18195
B4U62_RS17990
B4U62_RS16990
B4U62_RS16770
B4U62_RS16140
B4U62_RS16075
B4U62_RS14390
B4U62_RS14040
B4U62_RS13605
pstC
B4U62_RS13310
ypdA
B4U62_RS12450
B4U62_RS11410
B4U62_RS09525
