# Supplementary material for: Molecular Evolutionary Dynamics of Energy Limited Microorganisms
Source: Mol Biol Evol. 2021 Jul 13;38(10):4532–45. doi: 10.1093/molbev/msab195 (PMC8476154; doi:10.1093/molbev/msab195)
Supplement: msab195_Supplementary_Data [file msab195_supplementary_data.zip › supplement_latex.zip!/supplement_latex/figures/gene_convergence_plot_F.pdf]

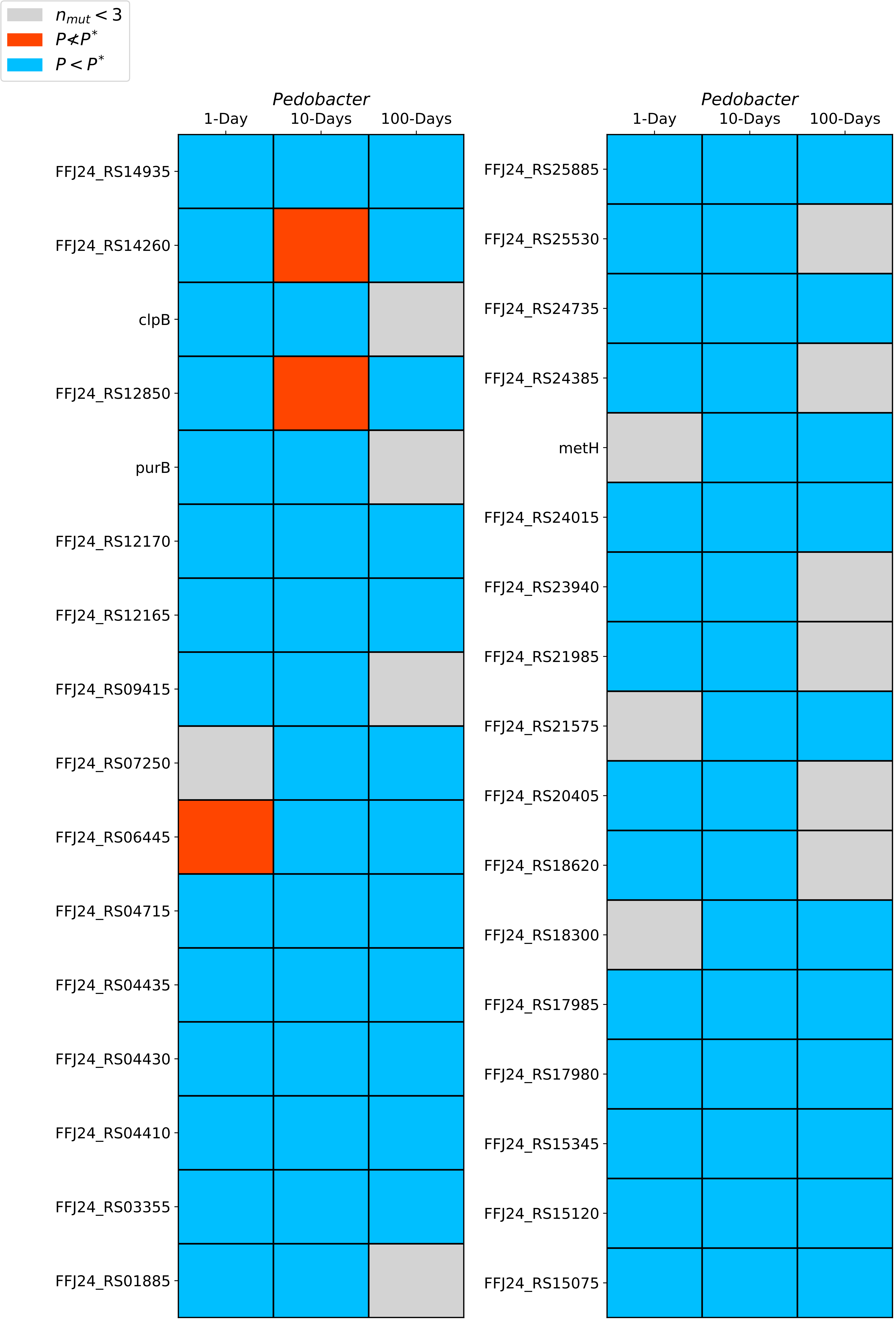

$n_{mut} < 3$
$P \nless P^*$
$P < P^*$
Pedobacter
1-Day
10-Days
100-Days
FFJ24_RS14935
FFJ24_RS14260
clpB
FFJ24_RS12850
purB
FFJ24_RS12170
FFJ24_RS12165
FFJ24_RS09415
FFJ24_RS07250
FFJ24_RS06445
FFJ24_RS04715
FFJ24_RS04435
FFJ24_RS04430
FFJ24_RS04410
FFJ24_RS03355
FFJ24_RS01885
Pedobacter
1-Day
10-Days
100-Days
FFJ24_RS25885
FFJ24_RS25530
FFJ24_RS24735
FFJ24_RS24385
metH
FFJ24_RS24015
FFJ24_RS23940
FFJ24_RS21985
FFJ24_RS21575
FFJ24_RS20405
FFJ24_RS18620
FFJ24_RS18300
FFJ24_RS17985
FFJ24_RS17980
FFJ24_RS15345
FFJ24_RS15120
FFJ24_RS15075
