# Supplementary material for: Molecular Evolutionary Dynamics of Energy Limited Microorganisms
Source: Mol Biol Evol. 2021 Jul 13;38(10):4532–45. doi: 10.1093/molbev/msab195 (PMC8476154; doi:10.1093/molbev/msab195)
Supplement: msab195_Supplementary_Data [file msab195_supplementary_data.zip › supplement_latex.zip!/supplement_latex/figures/gene_convergence_plot_P.pdf]

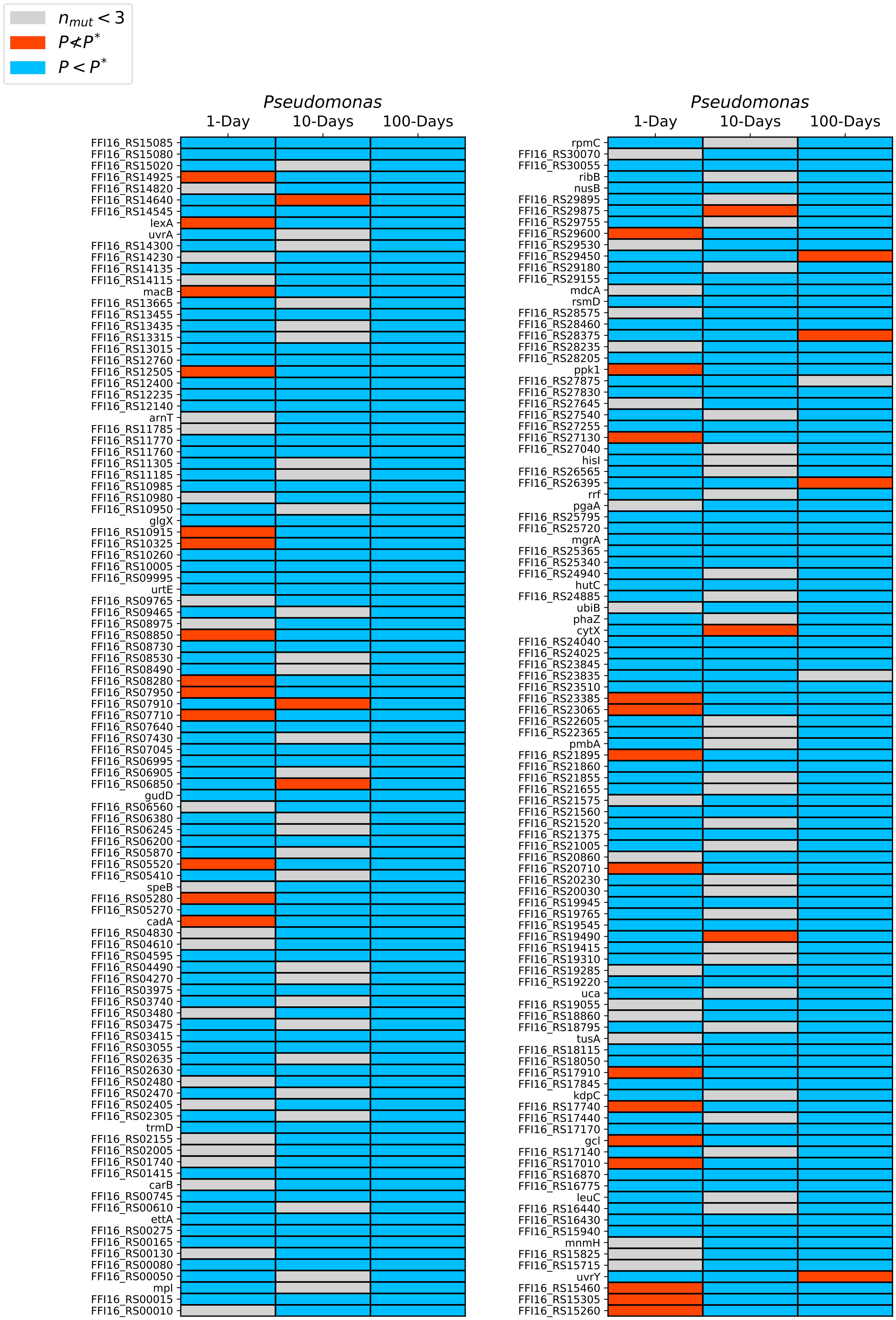

$n_{mut} < 3$
$P \nless P^*$
$P < P^*$
Pseudomonas
1-Day
10-Days
100-Days
FFI16_RS15085
FFI16_RS15080
FFI16_RS15020
FFI16_RS14925
FFI16_RS14820
FFI16_RS14640
FFI16_RS14545
lexA
uvrA
FFI16_RS14300
FFI16_RS14230
FFI16_RS14135
FFI16_RS14115
macB
FFI16_RS13665
FFI16_RS13455
FFI16_RS13435
FFI16_RS13315
FFI16_RS13015
FFI16_RS12760
FFI16_RS12505
FFI16_RS12400
FFI16_RS12235
FFI16_RS12140
arnT
FFI16_RS11785
FFI16_RS11770
FFI16_RS11760
FFI16_RS11305
FFI16_RS11185
FFI16_RS10985
FFI16_RS10980
FFI16_RS10950
glgX
FFI16_RS10915
FFI16_RS10325
FFI16_RS10260
FFI16_RS10005
FFI16_RS09995
urtE
FFI16_RS09765
FFI16_RS09465
FFI16_RS08975
FFI16_RS08850
FFI16_RS08730
FFI16_RS08530
FFI16_RS08490
FFI16_RS08280
FFI16_RS07950
FFI16_RS07910
FFI16_RS07710
FFI16_RS07640
FFI16_RS07430
FFI16_RS07045
FFI16_RS06995
FFI16_RS06905
FFI16_RS06850
gudD
FFI16_RS06560
FFI16_RS06380
FFI16_RS06245
FFI16_RS06200
FFI16_RS05870
FFI16_RS05520
FFI16_RS05410
speB
FFI16_RS05280
FFI16_RS05270
cadA
FFI16_RS04830
FFI16_RS04610
FFI16_RS04595
FFI16_RS04490
FFI16_RS04270
FFI16_RS03975
FFI16_RS03740
FFI16_RS03480
FFI16_RS03475
FFI16_RS03415
FFI16_RS03055
FFI16_RS02635
FFI16_RS02630
FFI16_RS02480
FFI16_RS02470
FFI16_RS02405
FFI16_RS02305
trmD
FFI16_RS02155
FFI16_RS02005
FFI16_RS01740
FFI16_RS01415
carB
FFI16_RS00745
FFI16_RS00610
ettA
FFI16_RS00275
FFI16_RS00165
FFI16_RS00130
FFI16_RS00080
FFI16_RS00050
mpl
FFI16_RS00015
FFI16_RS00010
Pseudomonas
1-Day
10-Days
100-Days
rpmC
FFI16_RS30070
FFI16_RS30055
ribB
nusB
FFI16_RS29895
FFI16_RS29875
FFI16_RS29755
FFI16_RS29600
FFI16_RS29530
FFI16_RS29450
FFI16_RS29180
FFI16_RS29155
mdcA
rsmD
FFI16_RS28575
FFI16_RS28460
FFI16_RS28375
FFI16_RS28235
FFI16_RS28205
ppk1
FFI16_RS27875
FFI16_RS27830
FFI16_RS27645
FFI16_RS27540
FFI16_RS27255
FFI16_RS27130
FFI16_RS27040
hisI
FFI16_RS26565
FFI16_RS26395
rrf
pgaA
FFI16_RS25795
FFI16_RS25720
mgrA
FFI16_RS25365
FFI16_RS25340
FFI16_RS24940
hutC
FFI16_RS24885
ubiB
phaZ
cytX
FFI16_RS24040
FFI16_RS24025
FFI16_RS23845
FFI16_RS23835
FFI16_RS23510
FFI16_RS23385
FFI16_RS23065
FFI16_RS22605
FFI16_RS22365
pmbA
FFI16_RS21895
FFI16_RS21860
FFI16_RS21855
FFI16_RS21655
FFI16_RS21575
FFI16_RS21560
FFI16_RS21520
FFI16_RS21375
FFI16_RS21005
FFI16_RS20860
FFI16_RS20710
FFI16_RS20230
FFI16_RS20030
FFI16_RS19945
FFI16_RS19765
FFI16_RS19545
FFI16_RS19490
FFI16_RS19415
FFI16_RS19310
FFI16_RS19285
FFI16_RS19220
uca
FFI16_RS19055
FFI16_RS18860
FFI16_RS18795
tusA
FFI16_RS18115
FFI16_RS18050
FFI16_RS17910
FFI16_RS17845
kdpC
FFI16_RS17740
FFI16_RS17440
FFI16_RS17170
gcl
FFI16_RS17140
FFI16_RS17010
FFI16_RS16870
FFI16_RS16775
leuC
FFI16_RS16440
FFI16_RS16430
FFI16_RS15940
mnmH
FFI16_RS15825
FFI16_RS15715
uvrY
FFI16_RS15460
FFI16_RS15305
FFI16_RS15260
