# Supplementary material for: Molecular Evolutionary Dynamics of Energy Limited Microorganisms
Source: Mol Biol Evol. 2021 Jul 13;38(10):4532–45. doi: 10.1093/molbev/msab195 (PMC8476154; doi:10.1093/molbev/msab195)
Supplement: msab195_Supplementary_Data [file msab195_supplementary_data.zip › supplement_latex.zip!/supplement_latex/figures/gene_convergence_plot_D.pdf]

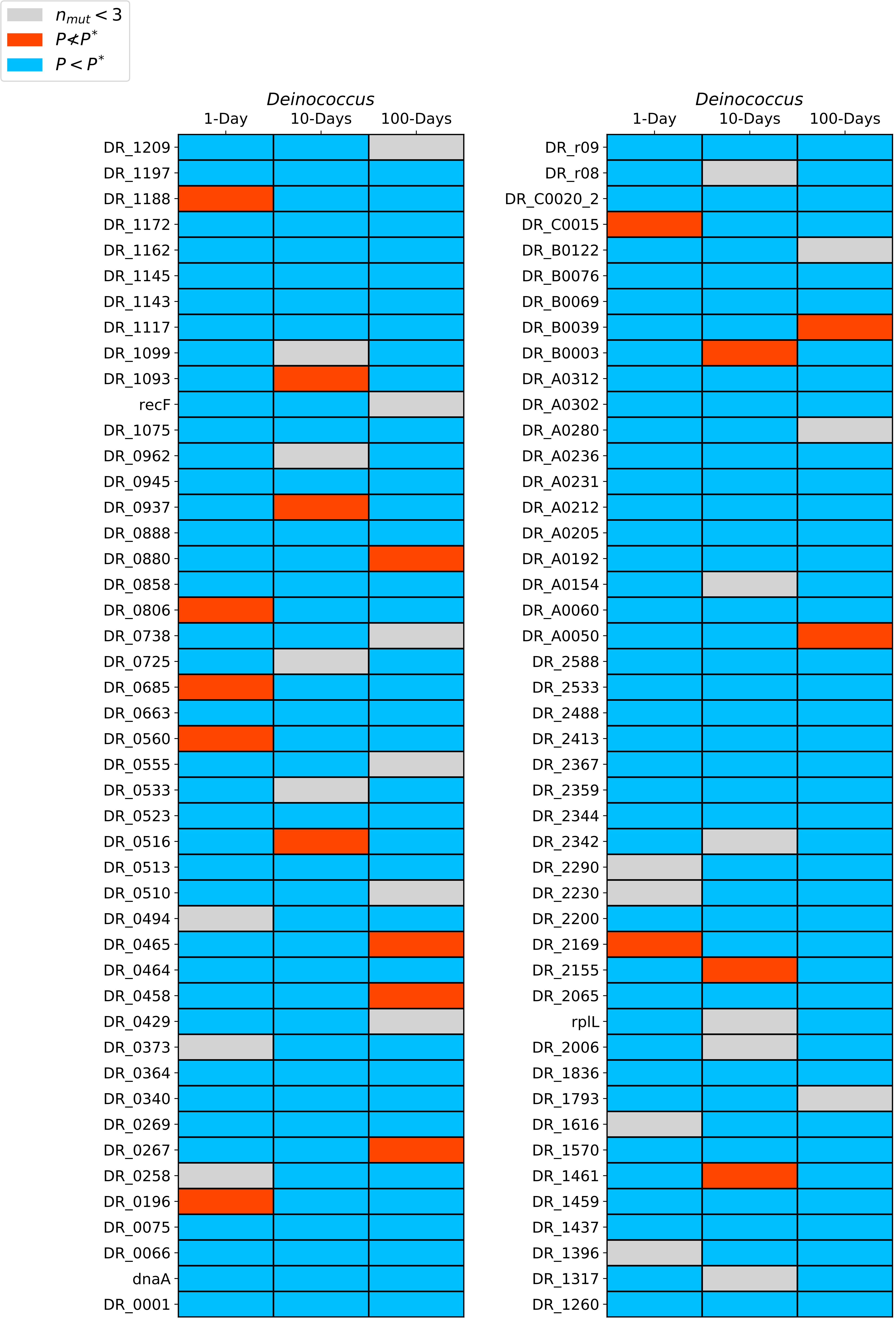

$n_{mut} < 3$
$P \not< P^*$
$P < P^*$
Deinococcus
1-Day
10-Days
100-Days
DR_1209
DR_1197
DR_1188
DR_1172
DR_1162
DR_1145
DR_1143
DR_1117
DR_1099
DR_1093
recF
DR_1075
DR_0962
DR_0945
DR_0937
DR_0888
DR_0880
DR_0858
DR_0806
DR_0738
DR_0725
DR_0685
DR_0663
DR_0560
DR_0555
DR_0533
DR_0523
DR_0516
DR_0513
DR_0510
DR_0494
DR_0465
DR_0464
DR_0458
DR_0429
DR_0373
DR_0364
DR_0340
DR_0269
DR_0267
DR_0258
DR_0196
DR_0075
DR_0066
dnaA
DR_0001
Deinococcus
1-Day
10-Days
100-Days
DR_r09
DR_r08
DR_C0020_2
DR_C0015
DR_B0122
DR_B0076
DR_B0069
DR_B0039
DR_B0003
DR_A0312
DR_A0302
DR_A0280
DR_A0236
DR_A0231
DR_A0212
DR_A0205
DR_A0192
DR_A0154
DR_A0060
DR_A0050
DR_2588
DR_2533
DR_2488
DR_2413
DR_2367
DR_2359
DR_2344
DR_2342
DR_2290
DR_2230
DR_2200
DR_2169
DR_2155
DR_2065
rplL
DR_2006
DR_1836
DR_1793
DR_1616
DR_1570
DR_1461
DR_1459
DR_1437
DR_1396
DR_1317
DR_1260
